# Women’s Experiences of Screening and Assessment of Mental Health Problems in the Perinatal Period: A Systematic Review and Meta-synthesis

**DOI:** 10.1007/s10567-026-00571-9

**Published:** 2026-05-08

**Authors:** Kerry Hozhabrafkan, Patricia Gooding, Anja Wittkowski, Sanaa Hyder, Sarah Peters

**Affiliations:** 1 Division of Psychology and Mental, School of Health Sciences, University of Manchester, Oxford Road, Manchester, M13 9PL UK; 2 Perinatal Mental Health and Parenting Research Unit, Greater Manchester Mental Health NHS Foundation Trust, Manchester, UK

**Keywords:** Perinatal mental health, Screening, Systematic review, Qualitative, Meta-ethnography

## Abstract

Mental health problems occur frequently in the perinatal period and timely identification is key for appropriate support to be offered. Women are routinely asked about their mental health and may undergo formal screening as part of healthcare services in the perinatal period. The aim of this review was to explore women’s views and experiences of being screened for mental health problems and to identify which aspects of this screening *process* they considered to be important. A systematic search was conducted across five electronic databases for potentially eligible studies published up to July 2025. A meta-ethnographic approach was used to synthesise the findings extracted from the thirty-eight papers included in the final analysis. Reciprocal translation resulted in a line of argument synthesis incorporating three over-arching themes. These were *‘Opportunities presented by screening’ ‘Difficulties with screening’ and ‘Context and constraints’*. Identification of salient aspects of the screening process revealed that preferences for how screening is conducted were varied, but more important than the specific method/mode used was the value that women placed on the concept of screening. Nevertheless, several process related factors, such as women’s knowledge and expectations of screening, the screening method and environment, were found to be influential. An important implication for clinical care is that perinatal mental health screening can offer impactful and positive opportunities for women when they see value in the process and when this is facilitated by well validated, person-centred, and flexible screening approaches. Questions regarding suicide/self-harm specifically, presented difficulties, and there is an urgent need to examine how this can be made more acceptable to women.

**Supplementary Information:**

The online version contains supplementary material available at https://doi.org/10.1007/s10567-026-00571-9.

## Introduction

The perinatal period, defined here as the duration of pregnancy and up to the first year postpartum, is a time when women may be particularly vulnerable to mental health problems because of the accompanying multitude of psychological, physiological, and life-style changes that they have to navigate. For example, depression is one of the most commonly experienced mood problems in both pregnancy and in the postpartum period, with a recent study reporting a global prevalence of 26.3% (Al-abri et al., 2023). Other common mental health problems experienced by women during pregnancy and/or in the postnatal period include anxiety and obsessive-compulsive disorder (OCD) (Fawcett et al., 2019; Salari et al., 2024). Furthermore, there is robust evidence that women who have pre-existing severe mental health problems (e.g., major depressive disorder, bipolar disorder or schizophrenia) are at risk of experiencing relapse and of developing severe conditions, such as postpartum psychosis (Alcantarilla et al., 2023; Taylor et al., 2018, 2019; Wesseloo et al., 2016). Of considerable concern, suicidal ideation is common in the perinatal period (Gelaye et al., 2016; Orsolini et al., 2016; Xiao et al., 2022), with suicide being a leading direct cause of death in women in the first year following the birth of a child. Suicide accounted for 16% of maternal deaths in the UK and Ireland between 2020 and 2022 (Allison Felker, 2024). Recent statistics from the United States show that 31.3% of pregnancy related deaths with mental health as an underlying cause were attributable to suicide (Policy Center for Maternal Mental Health, 2025). Looking more broadly, it has been reported that one in 100 maternal deaths across low- and middle-income countries was due to suicide; with the authors noting that this figure likely underestimates the true number, due to inconsistent classification and reporting of causes of maternal mortality (Fuhr et al., 2014). Furthermore, it has been estimated that up to 20% of postpartum deaths globally are due to suicide (Lindahl et al., 2005).

There is a large and growing body of research reporting associations between perinatal mental health problems and a range of adverse maternal and child outcomes (Aktar et al., 2019; Goodman, 2019; Howard & Khalifeh, 2020). These include increased rates of maternal morbidity, for example, gestational hypertension and antepartum haemorrhage, and adverse obstetric outcomes such as increased rates of caesarean section (Runkle et al., 2023; Rusner et al., 2016; Vigod et al., 2014). In addition, poor perinatal mental health has been shown to lead to compromised mother-infant interactions (Bernard et al., 2018; Ierardi et al., 2019) as well as adverse health and developmental outcomes in infancy and childhood (Hoffman et al., 2017; Pierce et al., 2020).

The mental health and wellbeing of women in pregnancy and beyond is now high on global public health agendas and is increasingly prioritised in the setting of policy and core standards of care for women in the perinatal period (Giridhar & MacDonagh, 2023). Moreover, recognition and assessment of mental health problems are now fundamental aspects of the role of health services, and the World Health Organization (2022) recommends that maternal and child health care providers should ask women about their well-being, as well as any stressors and depressive symptoms as part of usual care; stating that screening tools should be suited to the needs of perinatal women and adapted for local and culturally specific use. Detection of perinatal mental health problems via formal screening programmes in regions across the world is inconsistent, with varying programmes utilising different personnel and settings to assess mental health (Wilson et al., 2024). In the UK, National Institute for Health and Care Excellence (2014, 2016) recommend that women are asked about their mental health history during the first antenatal appointment and about their emotional wellbeing at every routine antenatal and postnatal contact, and in addition, that screening is carried out using a ‘case-finding’ approach utilising two depression case-finding questions (Whooley et al., 1997). In the case of a positive screen or clinical concern, then a screening instrument such as the Edinburgh Postnatal Depression Scale (EPDS) (Cox et al., 1987) or the Patient Health Questionnaire (PHQ-9) (Kroenke et al., 2001) are recommended.

The EPDS is the most widely used depression screening questionnaire in perinatal populations (Bhat et al., 2022; Gibson et al., 2009) with studies reporting validation across diverse samples and contexts (Hewitt et al., 2010; Sambrook Smith et al., 2022),. It is, of course, important that screening tools are well tested and contextually appropriate. However, to be satisfied that they offer a genuinely useful function, attention should be paid to ensuring that the methods used are perceived by women themselves to be both acceptable and helpful.

To date, three reviews have examined the acceptability of perinatal depression screening to women and healthcare professionals. Brealey et al. (2010) synthesised 16 qualitative and quantitative studies exploring views on postnatal depression screening with women and healthcare professionals. It was found that screening for postnatal depression was generally acceptable to both groups, generating insight into the specific aspects of screening that influenced participants’ views. For women, acceptability was linked to screening taking place at home, opportunity to have a conversation, the availability of feedback and being screened by a supportive and caring healthcare professional where reassurance and clarity was offered about their role. The review identified several areas that would benefit from further investigation, specifically, the acceptability of screening methods other than the EPDS and acceptability of screening where there is the potential for tools to either over or under-detect postnatal depression.

El-Den et al. (2015) expanded on the scope of this previous work by conducting a systematic review investigating the acceptability of screening for depression across the entire perinatal period. They found that most studies reported high acceptability of perinatal depression screening which used tools for both women and healthcare providers, and that a multitude of factors can influence acceptability. Two strengths of that review stood out in particular, namely: (i) Twenty-eight quantitative and qualitative studies were included that reported evidence about acceptability, and (ii) Numerous terms that participants used to express acceptability were described, underscoring its multi-faceted composition. However, across studies, the construct of acceptability was often poorly defined and operationalised and a wide range of diverse methods, from quantitative survey questions to in-depth interviews, were often adopted to examine acceptability (El-Den et al., 2015). A key issue identified by this review was that there is a need for agreed, objective criteria for assessing acceptability of screening for perinatal mental health problems.

A recent systematic review was particularly germane in the context of the rapid expansion of, and interest in, the development of digital health technologies to support perinatal mental health care (Novick et al., 2022; Singla et al., 2022). Clarke et al. (2024) evaluated qualitative, quantitative and mixed-methods studies to determine the extent to which digital screening for perinatal mental health problems was acceptable, feasible, and effective to both women and health care professionals. Twenty-nine studies were included in the analysis and the review found that digital methods were acceptable to women across different healthcare settings, countries, and cultures. The benefits described in relation to digital screening included providing an emotional outlet, the capacity for adaptation to specific language needs and facilitation of self-awareness and insight about one’s mental health. This review provides valuable insight into the acceptability of digital screening and the authors concur with the conclusion reached by Brealey et al. that we need to look beyond the EPDS in attempts to better understand screening acceptability, in addition to exploring the diverse needs of different populations of women.

Existing literature reveals many positive and negative perceptions of perinatal mental health screening, yet it also highlights how the concept of acceptability is complex, and evaluation of this as a discrete construct can be problematic. Therefore, a detailed exploration of personal perspectives of perinatal mental health screening is needed to better understand how different aspects of the entire screening *process* influence women’s views and experiences. Examples of potentially impactful components of the screening process range from the practical to the interpersonal and include (1) the contents of the screening measures, tools or questions (e.g., specific formats and question types), (2) settings in which screening takes place (e.g., clinic, the woman’s home, online or face-to-face), (3) explanations about the purpose of screening (e.g., mental health problems), and (4) interpersonal and communicative styles of the person doing the screening (e.g., empathic openness, cultural awareness, normalisation, offers of feedback, reassurance).

No systematic review to date has evaluated these four different components of the mental health screening process. Hence, our overall objective in conducting the current systematic review was to redress this gap, and to increase understanding of not only the different ways in which women view and experience perinatal mental health screening, but also to explain why they had come to hold these views. Qualitative research data provide an ideal medium through which to examine these different components of the screening process because they help to address both ‘how’ and ‘why’ questions (Pope & Mays, 2013). The research questions addressed by this systematic review were: (1) How did women perceive the process of being screened/assessed for mental health problems in the perinatal period? (2) Which aspects of the screening process were most salient to women?

## Methods

This systematic review and qualitative meta-synthesis was reported in line with Preferred Reporting Items for Systematic Reviews and Meta-Analysis (PRISMA) guidelines (Page et al., 2021) and eMERGe guidance (France et al. 2019a, b). The protocol was registered with PROSPERO on 22/06/2022 (Ref: CRD42022338746) https://www.crd.york.ac.uk/prospero/display_record.php?RecordID=338746).

### Search Strategy

Scoping searches were conducted using databases which were most relevant to this field of investigation. Five were subsequently selected for the purposes of this review (MEDLINE, CINAHL Plus, EMBASE, Midirs, and Web of Science. Using two published frameworks, namely, PICO (Richardson et al., 1995) and SPIDER (Cooke et al., 2012), a hybrid framework was developed to generate and organise search terms, incorporating the headings “Population”, “Intervention”, “Phenomenon” and “Evaluation” (PIPE). Medical Subject Headings (MeSH)/subject headings were utilised to identify additional indexed terms in individual databases. Boolean operators (“AND”, “OR”) were used to combine terms and concepts. Searches were limited to peer reviewed articles, but no date or language limits were applied. See Table 1 for the key terms used to operationalise each aspect of the review question and Online Resource 1 for details of the full search strategy used for each database.

Searches were conducted in May 2023 and updated in July 2025 prior to the final analysis. In addition, forwards and backwards citation searches of key papers was conducted. References identified from the search were managed using Endnote 20 software.

**Table 1:** Search terms used in systematic searches across all databases

Domain	Search terms
P—population	Perinatal OR Antenatal OR Pregnancy OR Postnatal AND
I—intervention	Assess* OR Screen* OR Disclos* OR Psychosocial Assessment AND
P—phenomenon	Mental Health OR Mental Illness OR Anxiety OR Suicid* OR Self-harm OR Depress* OR Perinatal Mental Illness AND
E—evaluation	Women’s Experiences OR Qualitative OR Qualitative Research OR Interview* OR Experience* OR Maternal Attitudes

*Limits applied*: peer reviewed articles. Search terms above were also combined with database specific MeSH terms/subject headings using “OR”

After duplicates were removed, KH screened the titles and abstracts of all articles for eligibility against the inclusion and exclusion criteria. Full texts of potentially eligible articles were read and categorised as “yes”, “no” or “maybe” in accordance with the criteria. A second reviewer (SH) read a randomly chosen 10% of all full text articles reviewed to assess them against the eligibility criteria. The level of agreement between reviewers was calculated using Cohen’s kappa and was excellent (91.7%, k = 0.84.) Any discrepancies were referred to a third reviewer (SP) and through joint discussion, resolution regarding inclusion was reached.

## Inclusion and Exclusion Criteria

Studies were eligible for inclusion if they (1) reported primary qualitative data (including mixed method designs if qualitative data could be extracted), (2) used a qualitative approach to analysis, (3) contained findings relating to women’s experiences of being screened or assessed for mental health problems/emotional distress in the perinatal period, (4) included data referring to the process of being asked and responding to questions about mental health in a healthcare context, and (5) were published in a peer reviewed academic journal.

Studies were excluded if they only reported findings relating to (1) the experience of perinatal mental health problems generally (e.g., describing symptoms or the experience of accessing support), (2) semantics when translating and validating screening instruments into another language, (3) the identification of factors that influenced help-seeking decisions/behaviours, (4) the experience of communicating about mental health outside of services (e.g., with partners, family members, on forums), and (5) conference abstracts, theses, position statements or other commentaries.

## Purposive Sampling

To ensure the data included would be sufficiently ‘rich’ to facilitate meaningful secondary analysis for a meta-ethnography (France et al. 2019a, b), and to balance this against including the experiences of as diverse a range of women and different screening contexts as possible, we then implemented purposive sampling. With the goal of including sources that provided rich data and maximum variation the following criteria was applied:

1. All papers were rated (between 1 and 5) on a scale of data richness (Ames et al., 2019) that takes into account the amount and quality of data that matches the synthesis objective. The scale used is presented as supplementary information Online Resource 2, Table S1.
2. Any paper scoring below an agreed cut-off threshold of 3 was then assessed as to whether it offered a novel perspective on the research question e.g., unique screening setting, method, minoritised participant population. If it did not, then it was excluded on this basis.

## Methodological Quality and Risk of Bias Assessment

Included studies were appraised for methodological quality/risk of bias using the 10-item Critical Appraisal Tools Programme (Critical Appraisal Skills Programme, 2018) (CASP) 10-item checklist for qualitative studies. This tool was chosen because it is widely used in health research (Long et al., 2020). In order to provide a summary overview and direct comparison of the papers, each item on the checklist was allocated a score based on the reviewer deciding “yes” = 1 point, “no” = 0 points and “unclear” = 0.5 points. Quality was then categorised as ‘low’ (0–5), ‘medium’ (6–8) or ‘high’ (> 8–10) (see Butler et al., 2020). CASP scores were not used to weight findings, nor were any studies excluded on the basis of the quality appraisal outcome.

## Data Extraction and Synthesis

The analysis was conducted using the meta-ethnographic approach outlined by Noblit and Hare (1988), which consists of seven phases (see Online Resource 3, Table S2). The approach was chosen because it facilitates the production of a novel interpretation of phenomena that can be used to produce new lines of argument and to inform theory generation (Sattar et al., 2021). Early identification of key papers in this domain revealed a source of conceptually rich data, and this is considered to be a necessary starting point for conducting a successful meta-ethnography (Flemming, 2022).

NVivo 12 software was used when conducting data extraction and coding. Copies of all papers were uploaded to the software and a database was created using ‘cases’ and ‘nodes’, which enabled us to file all of the study information and characteristics in one place but also to link each file to the corresponding data node trees. The raw data were extracted from each paper and coded with an independent node. Next, second order constructs from each paper were coded at the parent ‘extracted data’ node. Throughout this process, reading and re-reading of the papers was done to aide familiarisation.

Complete coding of the second order constructs (i.e. themes/concepts, remaining as close to the primary author’s definitions as possible) was then carried out, so that several code nodes for each paper were created. Next, similar concepts were placed into newly formed descriptive category headings. Through an iterative process of returning to the data under each category and making notes regarding similarities and differences, it was possible to merge these and new interpretations were formed. This process was undertaken through engaging with the raw data in NVivo, alongside the creation of data tables, lists and mind maps which facilitated analytical discussions within the review team.

### Reflexivity

It was important to acknowledge the review team’s positionality in relation to this research topic. All authors were female/women and some were mothers, all residing in the UK. Three were white European, one was of mixed heritage (white European/African-Caribbean) and one was south Asian. The first author (KH, a PhD researcher) was a mother and had a professional background in the healthcare professions working with this specific participant population and therefore had experienced perinatal mental health screening from ‘both sides’. SP and PG are experienced academic psychologists specialising in healthcare communication and suicide/mental health research, respectively. AW is an academic and practising clinical psychologist specialising in perinatal mental health problems, experienced in perinatal suicide assessment and research. SH is a trainee health psychologist and a PhD researcher, examining healthcare communication between doctors and patients from minority ethnic groups for her doctoral research. All members of the review team made conscious efforts to remain alert to their cultural, professional and sociodemographic positionality while conducting the synthesis. The ethnic diversity within the team was an asset in terms of challenging any implicit biases originating from euro-centric perspectives, and it facilitated rich discussions when considering our inclusion decisions for maximum variation sampling.

## Results

### Search Outcome

**Fig. 1:**
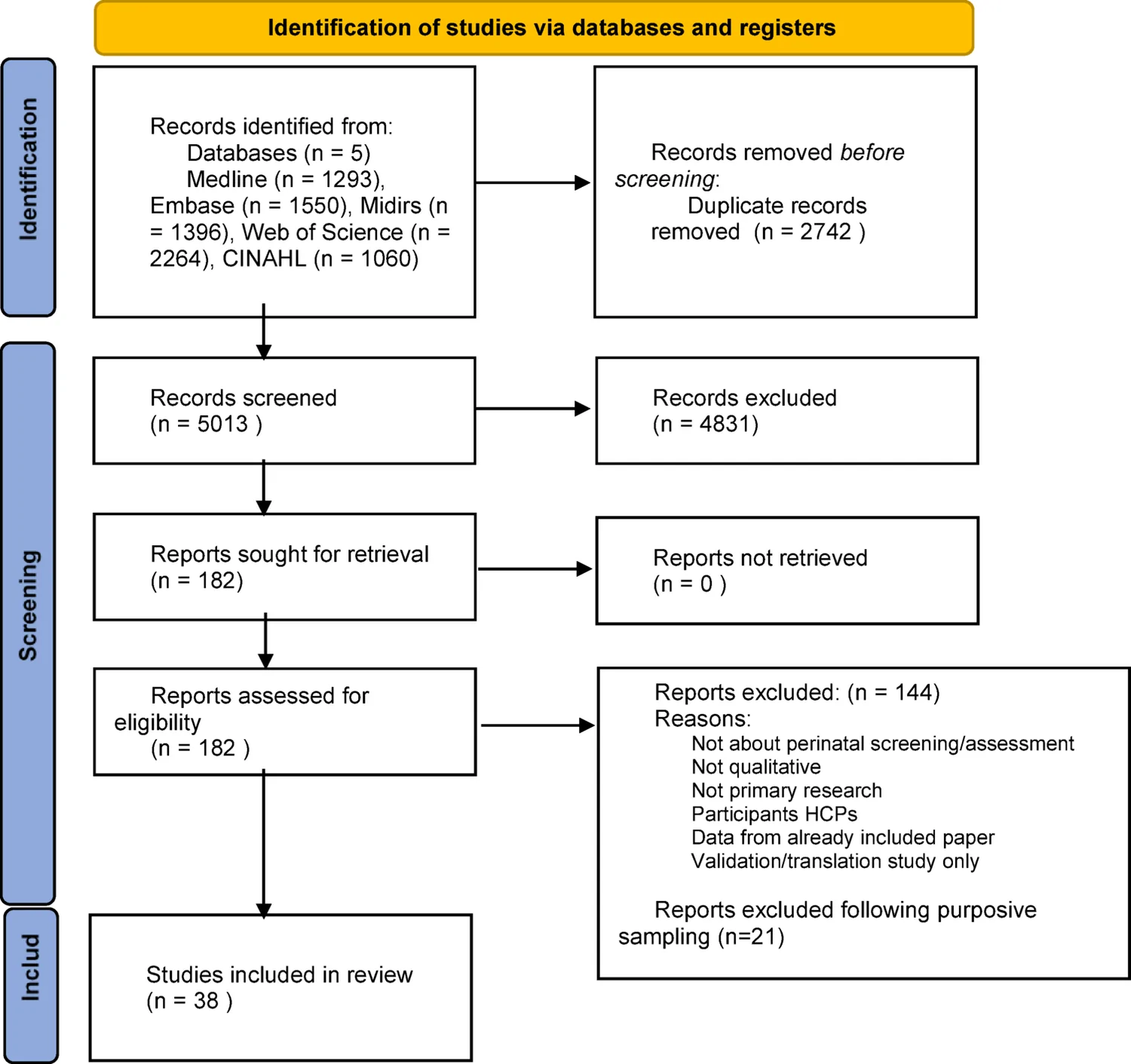
Prisma flow diagram showing searching and study selection. *From*: page MJ, McKenzie JE, Bossuyt PM, Boutron I, Hoffmann TC, Mulrow CD, et al. The PRISMA 2020 statement: an updated guideline for reporting systematic reviews. BMJ 2021;372:n71. https://doi.org/10.1136/bmj.n71

The search yielded 7563 records. After removing duplicates, 4872 titles and abstracts were screened against the inclusion/exclusion criteria. Full texts of 182 were assessed, of which 59 papers were eligible for inclusion. Next, the purposive sampling criteria was applied to these papers (see Online Resource 4, Table S3 for all papers that were excluded following purposive sampling). The final sample comprised 38 papers reporting findings based on the analysis of data collected in 35 studies. Figure 1 presents a summary of the process in a Prisma flow diagram. Papers were published between the years 2003 and 2025 and included qualitative and mixed method designs. Studies were conducted in the UK (*n* = 13), Australia (*n* = 8), the USA (*n* = 5), Canada (*n* = 2), Sweden (*n* = 1), South Africa (*n* = 1), Denmark (*n* = 1), Ireland (*n* = 1), India (*n* = 1), Kenya (*n* = 1), and New Zealand (*n* = 1). Seven of the studies focused on investigating perinatal mental health issues from the perspectives of women belonging to minority groups. These included women who were refugees, recent immigrants or a religious or ethnic minority to the country where the study took place, Aboriginal women, Maori women, pregnant adolescents and women living with HIV. Papers documented findings from antenatal (*n* = 352), postnatal (*n* = 334) and a combination of both antenatal and postnatal (*n* = 532) participants. Some of the studies (*n* = 9) included data reported from mothers with older children, ranging from 14 months up to 4 years, about their experiences during the perinatal period. Screening was conducted in both clinical and non-clinical settings and was conducted by a variety of people. These included: midwives, nurses, health visitors (UK), maternal and child health nurses (Australia), pharmacists, paediatric care providers, doctors and researchers. Some studies did not report who undertook screening. Table 2 provides detailed study characteristics.

**Table 2:** Characteristics of the 38 reviewed papers

	Study author, year, country	Study aim	Sample description	Screening method/context	Screener	Screening mode	Qualitative data collection and analysis method	Main themes/concepts
1	Witcraft et al. (2025) USA	To identify facilitators of mental health symptom and substance use endorsement and attendance to PMAD and PSUD treatment among Black PPP, in general and with the LTWP program.	Postnatal women (*n* = 68) Ethnicity: Black (100%) and Hispanic identity (3.5%)	Screening using a text/phone based screening and referral programme in a research context	Mobile phone based with feedback from a care coordinator	Text/phone	Interviews Grounded theory approach to qualitative content analysis	• Usability of the mobile-based tool • Comfort using the mobile-based tool • Necessity of the mobile based tool • Recommendations for the mobile-based tool
2	Kanan et al. (2025) USA	To explore the mental health experiences of Muslim American women in the perinatal period	Postnatal women (*n* = 18) Ethnicity: white, non-Hispanic (white) (83%) Hispanic Asian (17%)	Experiences of perinatal mental health screening generally Clinical setting	Non-specified	Not stated	Telephone interviews Thematic network analysis	• Need for tailored mental health support • Challenging screening experiences • Mental health experiences that affect maternal-infant bonding • Faith, culture and mental health
3	Hayward et al. (2025) New Zealand	To explore the perceptions of mental health screening in Wāhine Māori.	Postnatal women (*n* = 11) Ethnicity: Wāhine Māori.	General screening experience in a clinical context	Midwife	Not stated	Interviews Thematic analysis	• Historical impact • Relationships are paramount • Screening process • Issues with care
4	Dudeney et al. (2024a) UK	To assess acceptability and content validity of suicide items validated/used in perinatal populations	Antenatal and postnatal women (up to 2 years) (*n* = 21) Ethnicity: White British (*n* = 20), mixed (white Asian) (*n* = 1) Combination of women who had experienced mental health problems/suicidality and those who had not	Completion of 16 suicide related items from 7 screening measures Completed in a research context	Researcher	Remote	Cognitive interviews Framework analysis	• Item wording and content • Ambiguity of words and phrases • Concept definitions and the implications of nuance • Stigma, shame and judgment • The importance of context and framing
5	Dudeney et al. (2024b) UK	To explore the views of perinatal women about identifying/disclosing suicidality and HCPs about approaches to asking in maternity care settings	Same as in Dudeney et al. (2024a)	Same as in Dudeney et al. (2024a)	Researcher	Remote	Semi-structured interviews Thematic analysis (Braun & Clarke, 2006)	• Barriers to the identification and disclosure of perinatal suicidality: social and individual determinants • Barriers to the identification and disclosure of perinatal suicidality: determinants within the maternity care context • Approaches for identifying and discussing perinatal suicidality • Opportunities to facilitate the identification and disclosure of perinatal suicidality
6	Yuill et al. (2024) UK	To explore women’s views and experiences of assessment for perinatal anxiety	Antenatal and postnatal women (*n* = 41) Ethnicity: White Caucasian (93%), remainder not reported Lifetime mental health problems (*n* = 29)	Clinical Outcomes in Routine Evaluation (CORE- 10) Generalized Anxiety Disorder 7-item (GAD- 7), SAAS and Whooley questions. Completed in a research context	Non-specified clinical and researcher	Any	Semi-structured interviews Acceptability framework (Sekhon et al., 2017) and Thematic analysis (Braun & Clarke, 2006)	• Raising awareness and improving support • Surveillance and stratifying care • Personalizing care and building trust
7	Broberg et al. (2024) Denmark	To explore the experiences of pregnant women using the ANRQ and EPDS to identify psychosocial vulnerabilities in pregnancy	Antenatal women (*n* = 18) Ethnicity: Danish (*n* = 16), other ethnic origins (*n* = 2)	ANRQ and EPDS Completed in a research context	Researcher	Remote	Interviews Thematic analysis (Braun and Clarke, 2023)	• Feeling heard • An occasion for self-reflection
8	Fellmeth et al. (2023) India	To explore women’s awareness of perinatal mental health conditions, acceptability of being asked about mental health and preference for specific tools in two regions of India	Antenatal and postnatal participants (*n* = 29), non-perinatal participants (*n* = 7) Ethnicity: not explicitly reported but presumed Indian due to study objectives	Kessler Psychological Distress Scale (K10), EPDS, PHQ-9, GAD-7 and Perinatal Anxiety Screening Scale (PASS), PTSD Checklist (PCL-5), the Scale for Assessment of Somatic Symptoms (SASS) and the Suicide Behaviour Questionnaire Revised (SBQ-R) Completed in a research context	Researcher	Not stated	Focus groups discussions (*n* = 7) Thematic analysis (Braun & Clarke, 2006)	• Manifestations of perinatal mental health conditions and contributing factors • Challenges of talking about mental health • Acceptability of being asked about mental health
9	Pham et al. (2022) Australia	To explore perinatal women’s views of community pharmacist delivered postnatal depression screening and care	Antenatal and postnatal women (*n* = 41) Ethnicity: not reported	EPDS Asked in a research context about proposed screening by community pharmacist	Pharmacist	In person	Semi-structured interviews Themes mapped to Consolidated Framework for Implementation Research (Damschroder et al., 2009)	• Patient Experience with Existing PND Support and Screening Services • Familiarity with Pharmacists’ Roles • Pharmacist Visibility in PND Screening Care • Patient—Pharmacist Relationships
10	Mule et al. (2022) Australia	To assess pregnant women’s attitudes to and reasons for non-disclosure during psychosocial assessment with the midwife	Antenatal women (*n* = 161) Ethnicity: not reported	EPDS and ANRQ Completed in a clinical setting	Midwife	In person	Open ended question as part of online questionnaire Thematic analysis (Braun & Clarke, 2006)	• Normalising and negative self- perception • Fear of negative perception from others • Lack of trust of midwife • Women’s understanding and expectations of appointment • Mode of assessment and time issues
11	Chan et al. (2022) Australia	To explore the cultural validity of the EPDS, examining the perspectives of Aboriginal women and of midwives	Antenatal women (*n* = 13) Ethnicity: Aboriginal	EPDS Completed it a clinical setting	Midwife	In person	Yarning interviews Inductive thematic analysis and grounded theory	• Acceptability • Non-standardises EPDS • Strategies to increase engagement
12	Kohlhoff et al. (2021) Australia	To gain feedback from women regarding perspectives on antenatal mental health screening	Postnatal women (children aged 9–14 months) (*n* = 20) Ethnicity: Not recorded. Self-reported country of origin: Australia (*n* = 13), UK (*n* = 4), Brazil (*n* = 1), Hong Kong (*n* = 1), Malaysia (*n* = 1)	Mental health screening program delivered by midwives at a private hospital Completed in a clinical setting	Midwife	In person	Semi-structured interviews Thematic analysis (Braun & Clarke, 2006)	• Increased awareness and support for perinatal mental health issues • The program enhances the quality of care provided by the hospital • Experience with the midwife impacts perceptions of the program • Partners • Preparation for the appointment
13	Hsieh et al. (2021) USA	To understand how women from diverse backgrounds perceive the quality of perinatal depression screening and how this affects decisions about mental health care`	Antenatal and postnatal women (*n* = 29) Ethnicity: Black (*n* = 15) White (*n* = 11), Asian/Asian American (*n* = 2), Hispanic/Latina (*n* = 1), multiracial (*n* = 1)	EPDS Completed in a clinical setting	Non-specified clinical	In person	Interviews Thematic network analysis (Attride-Stirling 2001)	• Perceived ineffectiveness of the screening process • Provider’s disengagement with the depression screening • Macro-level barriers for depression screening
14	Willey et al. (2020) Australia	To determine if digital perinatal mental health screening is feasible and acceptable for women of refugee and migrant background	Antenatal women from a refugee background (*n* = 17) and migrant background (*n* = 5). Country of origin: Afghanistan (*n* = 10), Burma (*n* = 7), India (*n* = 3), Vietnam (*n* = 2)	Self-completion of the EPDS and psychosocial assessment tool using an IPad at the first antenatal visit Completed in a clinical setting	Midwife	In person	Semi-structured interviews and focus groups Hybrid approach to thematic analysis (Fereday & Muir-Cochrane, 2006)	• Women’s experiences of perinatal mental health screening in pregnancy • Barriers and enablers to accessing ongoing mental health care • Improvements to the program: the development of audio versions
15	Velloza et al. (2020) Kenya	To explore how perinatal women (half of whom were living with HIV) conceptualize depression and factors affecting depressive symptoms. To investigate acceptability of the PHQ-9 items to inform adaptation of the tool for depression screening in Kenya	Antenatal and postnatal women (*n* = 20), some living with HIV (*n* = 8) Kenyan	PHQ-9 Completed in a research context	Researcher	In person	Cognitive interviews (Willis, 2015)	• Item comprehension • Decision processes • Response processes • Suggested changes to the PHQ-9
16	Field et al. (2020) South Africa	To explore barriers and facilitators to mental health service access for pregnant adolescents in a low-resource setting	Antenatal adolescents (*n* = 12) aged between 15–19 years Ethnicity: not reported	Unspecified mental health screening questionnaire at the first antenatal visit	Midwife	In person	Semi-structured interviews. Framework analysis (Smith & Firth, 2011)	• Experience of mental health service use • Perceived barriers and facilitators to mental health service use • Preferred mental health service design.
17	Dyurich and Oliver (2020) USA	To explore the experience of using an electronic intervention app to screen for and manage symptoms of perinatal depression	Antenatal women (*n* = 6) Ethnicity: Hispanic/Latino (*n* = 3), White (*n* = 2), Asian/White (*n* = 1)	Completion of the EPDS using the ‘VeedaMom’ app. Completed in a research context	Self	Remote	In-app journaling, interviews and focus groups Descriptive phenomenology (Giorgi, 2009)	• Welcoming a new life: my own • Defining myself through comparisons • Need to address many emotions • Mommy-focused care • The high value of insight and acceptance • Apps are part of life • Benefits of the in-app EPDS • Teach me something new, now! •. . And please remind me
18	Doherty et al. (2020) UK	To examine design tensions surrounding use of a mobile app for self-report of psychological wellbeing when managing expectations and needs of pregnant women & HCPs	Antenatal women (*n* = 8 and mothers who had given birth in the last 4 years (*n* = 3). Ethnicity: White British (*n* = 3), White other (*n* = 4), White Irish (*n* = 1), mixed other (*n* = 2), Asian British (*n* = 1) Nationality: English (*n* = 5), Singaporean (*n* = 2), Greek (*n* = 1), Lebanese (*n* = 1), American (*n* = 1), French (*n* = 1)	Remotely sharing self-reports of psychological wellbeing and EPDS scores via the ‘BrightSelf’ app Completed in a research context	Self (data to midwife)	Remote	Design sessions Thematic analysis	• Perceptions of data sharing • Perceptions of the client/midwife relationship • Perceptions of data and its implications • Perceptions of wellbeing and the challenges of assessment • Perceptions of dialogue and feedback • Public health in society
19	Yapp et al. (2019) UK	To explore the acceptability of being asked about mental health problems at the booking appointment amongst a cohort of antenatal women	Antenatal women (*n* = 52) Ethnicity: White (*n* = 27), Black British (*n* = 6), Black Caribbean/African (*n* = 12), Asian/other (*n* = 7)	Whooley questions asked by Midwife Completed in a clinical context	Midwife	In person	Interviews Thematic analysis and framework approaches ( Attride-Stirling, 2001; Boyatzis, 1998; Braun and Clarke, 2006; Ritchie and Spencer, 1994)	• the experience of being asked about depression
20	Skoog et al. (2019) Sweden	To examine non-native-speaking immigrant mothers ‘experiences of screening for PPD	Postnatal women (*n* = 13) Nationality: Syria (*n* = 4), Kurdistan (*n* = 3), Iraq (*n* = 3), Kosovo (*n* = 2), Libya (*n* = 1)	EPDS completed in a clinical context (Child Health Service)	Nurse	In person	Interviews Latent content analysis (Elo & Kyngas, 2008)	• Challenging to speak about one’s mood • Feeling confirmed as a person • Reminding of what is lost and possibilities to come
21	Carlin et al. (2019) Australia	To explore the acceptability of the KMMS in perinatal health care	Antenatal women (*n* = 9), mothers (*n* = 6) Ethnicity: Aboriginal from the Pilbara region	EPDS and KMMS	Researcher	In person	Yarning interviews Thematic analysis	• A lived experience • Connectedness and support • Yarning safely
22	Nagle and Farrelly (2018) Ireland	To examine women’s experiences of having their mental health needs considered in the perinatal period	Postnatal women (*n* = 8) Nationality: Irish (*n* = 6), Eastern European (*n* = 1), Other (*n* = 1	General enquiry about mental health in a clinical setting	Midwife	In person	Interviews Thematic analysis (Braun & Clarke, 2006)	• The experience of mental distress • Telling and disclosing • The experience of obtaining help
23	Marsay et al. (2018) South Africa	To explain the mechanisms and circumstances by which measurement reactivity occurred in antenatal women who participated in a screening interview	Antenatal women (*n* = 55) Ethnicity not reported	EPDS, Whooley questions and NetSCID in a clinical setting	Doctor/researcher	In person	Narrative inquiry interviews Themes (Neuman et al., 2000)	• Talking helps: disclosure • Anonymity • Putting words to feelings: validation of experience • Gaining self-knowledge • Agency
24	Littlewood et al. (2018) UK	To investigate the acceptability and impact of the Whooley and EPDS depression case-finding questions and the extent to which they capture appropriate information for effective screening/case-finding of depression in perinatal care	Antenatal and postnatal women (*n* = 25) serial interviews Ethnicity not reported	EPDS & Whooley questions completed in a research context	Researcher	In person	Interviews using a cognitive framework Framework analysis (Ritchie & Spencer, 2002)	• Acceptability of screening/case-finding • Ease of understanding Whooley and EPDS • Ease of remembering Whooley and EPDS • Confidence in answers to Whooley and EPDS • Comfort to answer Whooley and EPDS • Overall comparisons of Whooley and EPDS • When is feeling tired and hopeless a problem in the perinatal period? • Social expectations of pregnancy and motherhood • Facilitators of, and barriers to, screening/case-finding
25	Doherty et al. (2018) UK	To explore the challenges and issues in using mobile phones for self-report of psychological wellbeing during pregnancy	See Doherty et al. (2020)	See Doherty et al. (2020)	Self (data to midwife)	Remote	Design sessions Themes	• The pregnancy journey • Experience of perinatal care Technology use in pregnancy
26	Evans et al. (2017) UK	To explore women’s experience of anxiety in pregnancy and views on the use of anxiety instruments in antenatal care.	Postnatal women (*n* = 19) Ethnicity not reported	Edinburgh Postnatal Depression Scale (EPDS, Cox et al., 1987), the Hospital Anxiety and Depression Scale (HADS, Zigmond & Snaith, 1983) and the Pregnancy Related Anxiety Questionnaire (PRAQ, Van den Bergh, 1990) in a research context	Researcher	In person	Focus groups Template analysis (King, 1998)	• Sources of support • Administration of anxiety instruments • Instruments prompting discussion
27	Bayrampour et al. (2017) Canada	To understand women’s perspectives of different mental health screening approaches and the perceived barriers to the communication and disclosure of their mental health concerns during pregnancy.	Antenatal women (*n* = 15) Ethnicity: White Caucasian (*n* = 11), Other (*n* = 4)	Unspecified method in a clinical context	Non-specified clinical	Any	Interviews Thematic analysis (Boyatzis, 1998)	• Less interactive approach • Communicative approach • Internal barriers • Not recognizing the symptoms • Not understanding the emotions • Fear of being judged • Fear of the consequences • Structural barriers
28	Williams et al. (2016) UK	To explore midwives and women’s experiences of the three depression case-finding questions at the booking appointment	Antenatal women (*n* = 20) Ethnicity: White British (*n* = 14), White other (*n* = 2), Asian (*n* = 2), Black-African (*n* = 2)	Whooley questions in a clinical context	Midwife	In person	Interviews Thematic using a constant comparison approach (Glaser et al., 1968)	• Mental health discussion in consultation • Reasons to confide or not confide in midwife • Women’s views on being asked the three depression case finding questions
29	Darwin et al. (2016) UK	To provide validation of the Whooley and Arroll questions at booking and explore women’s views and experiences of antenatal mental health assessment using these	Antenatal and postnatal women (*n* = 22) serial interviews Ethnicity: White British (*n* = 17)	Whooley and Arroll questions and EPDS in clinical and research context	Midwife	In person	Interviews Framework analysis (Ritchie & Spencer, 1994)	• Context of disclosure • Context of maternity appointments • Understandings of maternal mental health • Purpose of assessment and implications of disclosure
30	Mann et al. (2015) UK	To examine the acceptability of case-finding questions to identify perinatal depression	Antenatal and postnatal participants (*n* = 152) Serial time points Ethnicity: White British (*n* = 81), White other (*n* = 5), Mixed (White & Black) (*n* = 4), Mixed (White & South Asian) (*n* = 3), Black (*n* = 6), Indian (*n* = 5), Pakistani (*n* = 38), Bangladeshi (*n* = 5), Other (*n* = 5)	Whooley questions in a research context	Researcher	In person and postal	Intra-method mixing survey (Johnson & Turner, 2003)	• General acceptability comment • Completing and answering the questions • The value of asking the questions • Expressing own personal feelings
31	Coates et al. (2015) UK	To explore the symptoms of mental health problems reported by new mothers and their experiences of being assessed for these	Postnatal women (*n* = 17) Ethnicity: White European (*n* = 16), Chinese (*n* = 1)	Not specified (some participants talked about the EPDS)	Not specified Clinical	In person	Interviews Thematic analysis (Braun & Clarke, 2006)	• Not identifying with postnatal depression • Need to normalize support seeking • Need for support irrespective of diagnosis • Importance of timing • A questionnaire is not sufficient
32	Rollans et al. (2013) Australia	To describe women’s experiences of psychosocial assessment and depression screening and examine the meaning they attribute to assessment and how this influences their response	Antenatal and postnatal women (*n* = 34) Serial time points Nationality: Born outside Australia – England/Ireland (*n* = 5), non-English speaking countries i.e. Laos, Egypt, India, China (*n* = 15)	EPDS and psychosocial assessment in a clinical context	Midwife and Child Health Nurse	In person	Observations and interviews Descriptive analysis using frequencies and Thematic analysis (Braun & Clarke, 2006)	• Unexpected - ‘a bit out of the blue’ • Intrusive: very personal questions • Uncomfortable: ‘digging over that old ground’
33	Darwin et al. (2013) UK	To illustrate measurement reactivity in the perinatal period and consider the mechanisms through which it occurs, drawing on differences between assessment methods, settings and individuals.	See Darwin et al. (2016)	See Darwin et al. (2016)	Clinical and research settings	In person	Interviews	• Reactivity is reduced when assessment is perceived as routine • Reactivity occurs through self-appraisal • Reactivity occurs through validation of an individuals’ experiences and is influenced by the assessor’s reactions • Reactivity occurs when assessment acts as a listening visit • Reactivity occurs when the assessment induces the individual to seek support
34	Byatt et al. (2013) USA	To identify factors that impede women from seeking help for PPD in a pediatric setting and facilitators to detecting and addressing PPD in this setting	Postnatal women and mothers (*n* = 27) Ethnicity: White (*n* = 27)	Any screening in a clinical setting	Pediatric care provider	In person	Focus groups Grounded theory (Morgan, 1988)	• Ambivalence about screening due to concerns about stigma and losing parental rights • Ambivalence about the role of pediatric health providers screening • Pediatric health care professionals are not trained to screen or discuss PPD • Addressing both medical and mental health care needs of mother and baby • Therapeutic, de-stigmatizing approach to screening
35	Armstrong & Small (2010) Australia	To explore how women view and experience being screened for postnatal depression	Postnatal women (*n* = 147) Ethnicity not reported	EPDS in a clinical context	Maternal and child health nurse	In person	Postal surveys (*n* = 147), telephone contacts (*n* = 80) and interviews (*n* = 20)	• The EPDS as a “good springboard for conversation” • Stigma: “why did she give it to me?” • Timing and responsiveness • The EPDS as a “test” • A window of opportunity to talk
36	Godderis et al. (2009) Canada	To present the results of a study that used cognitive interviewing techniques to interview pregnant and postpartum women about their experience of completing the Edinburgh Postnatal Depression Scale	Antenatal (*n* = 4) and postnatal women (*n* = 4) miscarried (*n* = 1) Ethnicity: Caucasian (*n* = 2), did not identify with a particular ethnicity (*n* = 7)	EPDS in a clinical context	Researcher	In person	Cognitive interviewing techniques	• Item 3: I have blamed myself unnecessarily when things went wrong • Item 4: I have been anxious or worried for no good reason • Item 6: Things have been getting on top of me • Item 7: I have been so unhappy that I have had difficulty sleeping • Item 9: I have been so unhappy that I have been crying • Item 10: The thought of harming myself has occurred to me
37	Poole et al. (2006) UK	To provide information on women’s views on being screened for postnatal depression	Postnatal women (*n* = 15) Ethnicity not reported	EPDS in a clinical context	Health Visitor	In person	Interviews Interpretative phenomenological analysis	• Context • Expectations of screening • Disclosure • Interpersonal relationships • Relevance of the screening tool • Getting the results • Transition to motherhood
38	Shakespeare et al. (2003) UK	To explore the acceptability to women of postnatal screening by health visitors with the EPDS	Postnatal women (*n* = 39) Ethnicity: White (*n* = 37), Other (*n* = 2)	EPDS in a clinical context	Health Visitor	In person	Interviews Constant comparative	• Problems with the process of screening • Personal intrusion of screening • Stigma

### Risk of Bias Assessment

Thirty of the studies were categorised using the CASP as high quality and 8 as medium quality. None of the included studies were judged to be of low quality. A second team member (SH) appraised all studies independently. Results were compared during discussions regarding rationale for individual quality ratings, and 89.47% agreement was reached. The ratings and appraisal comparisons can be viewed in Online Resource 5, Table S4.

### Meta-Synthesis

**Fig. 2:**
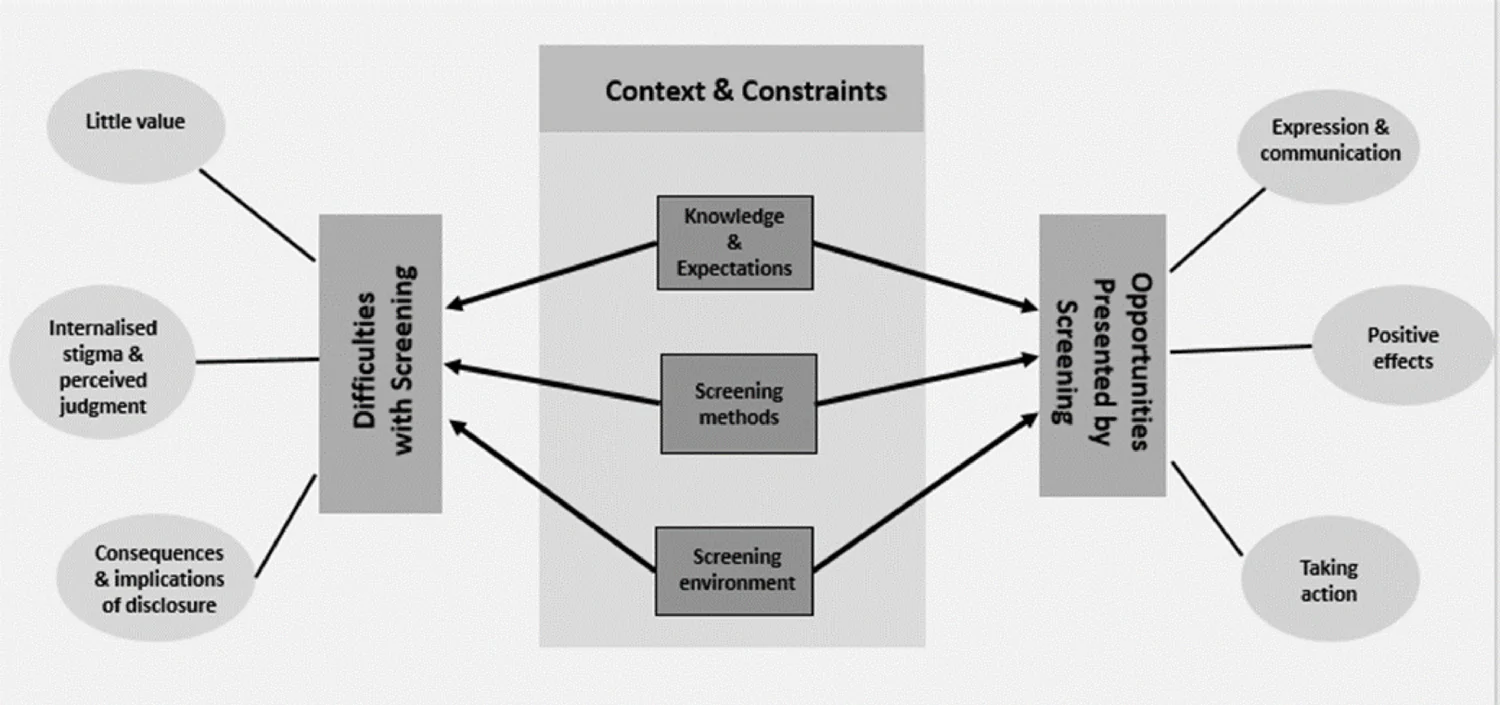
Conceptual model showing interrelated themes and constructs

Reciprocal translation of the papers resulted in the identification of three, inter-related main themes which each comprised a number of constructs that describe them in greater detail. Together these culminated in a line of argument synthesis (Noblit & Hare, 1988) which posits that perinatal mental health screening was perceived as a process that presented at one end of a continuum opportunities, and at the other end a concatenation of difficulties. Several contextual and constraining factors were found to influence the balance of these views for women. The main themes were: ‘Opportunities presented by screening’ ‘Difficulties with screening’ and ‘Context and constraints’. Findings (known in meta-ethnography as the third order constructs) are presented below with illustrative participant quotes (known as first order constructs). Table 3 provides examples of how the analysis process was developed using the primary authors’ interpretations of the data (known as second order constructs) and how these, informed the development of our new third order constructs i.e. the final thematic structure. A visual conceptual model (Fig. 2) depicts the lines of argument synthesis which demonstrates the relationship between the themes and their associated constructs.

**Table 3:** Reciprocal translation examples

Third-order constructs (reviewers interpretations based on secondary analysis of first and second order constructs)	Second-order constructs (primary author’s interpretations of the participant data)	First-order constructs (illustrative participant quotations from primary studies)
*Opportunities*		
Enabling expression and communication	Helping to talk it through	*“Sometimes it’s just multiple choice and none of them really feel like they fit and I don’t want to have to find an answer or to write it to explain myself either it becomes really long or I’m not sure that I’m getting my point across. So I think talking to someone verbally whether it’s on the phone or in person – there’s no difference – at least I feel confident that they get my point across and they can properly evaluate.”* (Bayrampour et al., 2017)
To feel positive effects	High value of insight and acceptance	*“So I had to kind of take a step back and think what am I doing? What’s going on?…[depression] happens without you knowing it”* (Dyurich & Oliver, 2020)
To take action	Screening as an intervention	*“I think what triggered getting everything sorted out was actually talking to you about it. … And I actually went and spoke to a counsellor after I had spoken to you and it’s the most liberating thing I’ve ever done.” (*Darwin et al., 2013)
*Difficulties with screening*		
Little value in screening	Views on the meaning of the help question	*“To be honest I don’t think anybody could really do anything to help because it’s just the situation and I just need to sort it out myself”* (Williams et al., 2016)
Internalised stigma and perceived judgment	Fear of negative perception from others	*“I was embarrassed about the answers and was concerned it would project me in a poor way.”* (Mule et al., 2022)
Consequences and implications of disclosure	Disclosure	*“My main thing I was so frightened of was that they would take the baby off me.”(*Poole et al., 2006)
*Context and constraints*		
Knowledge and expectations of screening	Preparation for the appointment	*“When I booked in*,* if there could even be like*,* “These are the things we’re going to talk about*,* and it’s a really safe space and this is why you could have your partner or not have your partner.” (*Kohlkoff et al., 2021)
Screening methods	The wording of the Whooley questions	*“I definitely felt a little bit down but… it was very like black or white*,* ‘yes’ or ‘no’ kind of thing whereas I kind of felt like I was more in a slightly grey area.”* (Yapp et al., 2019
Screening environment	Contextual constraints of antenatal booking	*“It was all a little bit rushed*,* and*,* you know*,* there’s a lot to fit into that*,* which is not her [midwife] fault*,* but*,* kind of how the system works. You can see that. But… I just felt a bit… It wasn’t that satisfying really.”* (Yapp et al., 2019)

One example representing each construct is provided

### Main Theme 1: Opportunities Presented Through Screening

This theme represents the positive aspects of screening reported in the studies. When participants viewed screening as an acceptable, worthwhile, and helpful endeavour, opportunities to experience personal benefit were perceived. The constructs of ‘enabling expression and communication’, ‘experiencing positive effects’, and ‘taking action’ describe these opportunities in further detail.

### Enabling Expression and Communication

Screening was viewed as an opportunity for women to express how they were feeling and to communicate their concerns. Women reported seeing screening as a catalyst for conversation and for some, this acted as an emotional outlet, providing an experience that brought a sense of relief and catharsis.

*“You know*,* at least you asked me and it cancelled all the pain*,* because I don’t even talk to someone but the time I talked to you*,* when I got at home I felt so happy that I took everything out to someone”* (Marsay et al., 2018).

Screening could facilitate an exchange of information which allowed women to properly articulate their distress, provide necessary context and feel understood. The screening process being experienced as a two-way communication process was extremely important to women, as this facilitated being able to both give and receive clarification as well as to gain feedback and reassurance. For many participants, this meant having a conversation with the person administering the screening in addition to any screening questionnaires they might complete.

*“The opportunity to elaborate and explain would be more helpful in the context of establishing wellbeing and emotional support needs.”* (Mule et al., 2022).

### Experience Positive Effects

Screening presented an opportunity to feel validated and to gain recognition and insight regarding participants’ mental health. Through screening, women were better able to understand what they were experiencing - specifically within the context of being pregnant or postnatal, and to recognise the extent to which these feelings could be indicative of a mental health problem.

*“It was the first time that I could find a descriptive word that could match how I was feeling. It was like ‘panicky to take him somewhere or drive him somewhere’ and just in general checking up around him during the night and things like that… I recognized that I was panicky versus concerned then I realized that I had you know my feelings were coming from a place of fear versus a place that’s probably more normal.”* (Bayrampour et al., 2017).

Being questioned about suicidal feelings was particularly impactful in terms of facilitating participants’ to ‘give voice’ internally to what they may be experiencing. This was especially important where participants had struggled to understand and ascribe meaning to their thoughts, for example, realising that their thoughts were about suicidality.

*“I think for myself*,* and maybe a lot of other people*,* it’s confusing… especially around suicidality*,* the thoughts are so abstract*,* so it’s not until you actually answer the question*,* like until you actually sort of say out loud*,* that you’re like*,* ‘oh*,* shit’… and that feels to me like a really important step in the sort of process of reflecting and getting help”.* (Dudeney et al., 2024)

Gaining new insight into their feelings came with a sense of relief and validation for some women. Validation occurred when women felt able to express their distress, often for the first time, and to have this acknowledged and understood. This led participants to reflect on their own strengths and resilience, rather than viewing poor mental health as a failure. Such re-appraisal led to an increase in self-compassion and acceptance.

*“I was reading through the questions thinking that lots of women may be having the same experiences*,* similar thoughts*,* and it just makes you feel like well it’s not you either*,* you know*,* being weak or not being able to deal with things*,* it’s quite natural. … it just makes you feel like that you’re not going bonkers.”* (Darwin et al., 2013).

Even for women who were not experiencing mental health problems, the very act of being screened highlighted in the moment, the importance of monitoring mental health, serving to increase general awareness and to normalise struggles. For those who were experiencing mental health difficulties, it meant feeling less alone in their struggles.

*“There was definitely a sense of relief in answering*,* and you could see that*,* okay*,* it’s okay to write that it’s a bit difficult—I mean*,* it’s probably not just me who thinks it’s difficult if it’s written here” *(Broberg et al., 2024).

For many participants, screening meant feeling cared for and they relayed being comforted by the interaction, not only because of the normalising effect of the questions, but also because of the connection they experienced with the screener on an interpersonal level. This was particularly notable for women belonging to marginalised populations, such as the study conducted with Kenyan participants who were living with HIV.

*“The questions make me feel at peace. They are a source of comfort. It makes me feel that I am not alone in this because when I am given this question*,* I know there are others who also have [depression] and that is comforting. You (the interviewer) are also a source of comfort.” *(Velloza et l., 2020).

### Taking Action

Screening often acted as a positive intervention, in addition to conferring these other positive benefits, because women reported being induced to take action as a result of discussing their mental health during screening. This took the form of confiding in others and seeking help with their mental health problems independently, as a result of the screening process.

*“When I showed my husband the letter and then I think maybe he realised maybe he must be more there. And then whenever I had to talk*,* I can talk to him. Ja. He actually felt bad*,* you know. Ja*,* he thought he was not there enough. So then*,* but now he is very supportive.”* (Marsay et al., 2018).

Whilst the prospect of accessing help and support from health services motivated many women to engage with screening processes, for some, the process itself inspired a sense of positivity, agency and impetus to proactively address mental health concerns.

*“I’m suffering from something*,* I should do something about it”* (Doherty et al., 2018).

### Main Theme 2: Difficulties with Screening

This theme describes the negative views and experiences of screening or assessment that were expressed by women. These were characterised by the constructs ‘Little value in screening’, ‘Internalised stigma and perceived judgment’, and ‘Consequences and implications of disclosure’.

### Little Value in Screening

Whilst some women found that screening aided personal recognition of mental health problems, others were unable to differentiate between ‘normal’ and ‘abnormal’ feelings in the perinatal period and felt screening offered little to help them achieve this differentiation. This meant that women saw minimal benefit in being screened as they struggled to understand themselves or to communicate about their distress in this context.

*“I think I was just so emotional that it was just better to keep it all inside and figure out and try to understand what was going on and try to understand … I just felt that like my emotions were all over the place*,* all the time. And I just didn’t understand them. I’m probably still like that.”* (Bayrampour et al., 2017).

Women felt that screening instruments did not adequately take into account the fact that they were navigating the transition to motherhood, and that transient distress might be expected. Furthermore, normalisation of difficult emotions in the perinatal period due to, for example, hormonal changes, or participants attributing poor mental health to wider life circumstances, created a sense of ambivalence about screening.

*“When you are pregnant you respond to stressful situations differently*,* compared to when you are not. I just put it down to a normal part of getting used to such big life changes and hormones.” *(Mule et al., 2022).

Participants expressed negative views about screening when it did not seem worthwhile to them. This occurred when participants suspected that the healthcare professional did not really care about the answers given to screening questions and there was a lack of feedback given; or when there was a perception of screening acting as a means of gatekeeping access to treatment. Women anticipated that they would not receive access to a form of support that would be genuinely helpful as a result of screening. They often navigated the process of answering screening questions using something akin to a ‘cost-benefit analysis’ approach, whereby the potential gains were weighted against the perceived downside of being open about their mental health and fully engaging with screening.

*“For me*,* it’s to almost like a qualification to get in to access certain services basically. I feel like if I haven’t scored ‘high enough’ on the questionnaires*,* I would’ve not been referred to the perinatal mental health team… If you score low*,* you’re seen by the community midwives; if you score like middling*,* you’re seen by perinatal mental health midwives; if you score high*,* you’re seen by the perinatal mental health team*,* so it’s almost like kind of gatekeeping in a way. It will only let the most eligible people into certain services.”* (Yuill et al., 2024).

The belief that mental health was beyond the remit of maternity services and/or specific health professionals, or that such individuals were inadequately equipped to address mental health concerns and emotional distress, was also conveyed by participants. Consequently, participants anticipated that they would not receive access to adequate support, rendering the screening futile.

*“I didn’t feel my needs would be met by her handing me a flyer or referring me somewhere. It was clear to me that she had very limited knowledge of anxiety or the medicine I take for my condition.”* (Mule et al., 2022).

Finally, some women found the process of being asked about their past and current mental health intrusive and possibly irrelevant to their perinatal status, preferring to seek help for a mental health problem independently. A lack of confidence in maternity healthcare professionals was evident in several women’s accounts, as they expressed views that midwives, for example, lacked competence and training to respond appropriately to any mental health concerns.

*“A midwife is not a mental health professional”* (Doherty et al., 2020).

### Internalised Stigma and Perceived Judgment

Women viewed screening as difficult to navigate because they experienced many self-directed negative feelings such as fear, shame, and guilt in association with their mental health difficulties. Where the concept of recognition of a mental health problem was a positive opportunity for some, for others the prospect of ‘seeing’ and acknowledging their distress to themselves due to screening felt exposing and confronting.

Women talked about the pervasive stigma surrounding mental health problems – particularly in the perinatal period - and how this caused them to approach screening with, at best, caution and, at worst, fear. Women referenced the societal expectation that women should feel joyful and delight in their role as expectant or new mothers. This notion of idealised motherhood was intrinsic to such internalised stigma. Fear of judgment from others (especially healthcare professionals) and of being labelled as ‘mentally ill’ or an unfit parent was prominent in women’s accounts.

*“There is a stigma that goes along with mental health…you know sick patients take on a sick patient role*,* I didn’t want to take on that role - this ‘mental health patient’ role. I don’t like to feel like I’m being boxed off in that ’tragic’ box.”* (Nagle & Farrelly, 2018).

Participants were reluctant to talk about suicidal cognitions and experiences during screening, fearing it was an unacceptable thought for a mother to disclose.

*‘‘You feel like you’re crazy when you do that [have thoughts about harming yourself]. It is admitting something is really wrong—you’ve passed the line. You’ve been taught never to think like that.’’* (Godderis et al., 2009).

These feelings were rooted in socio-cultural stigma and were often amplified for women from minority ethnic groups. They experienced additional challenges such as racism and beliefs related to their religion or culture that could conflict with openness and addressing the need for mental health support for women. This is clearly exemplified by the following quote taken from a study that sampled women living in two different regions of India.

*“A person wants to speak his heart*,* but our society is such that it will call him a lunatic. Everyone makes fun of him. They just become a laughter stock*,* the people who talk about such things.”* (Fellmeth et al., 2022).

#### Consequences and Implications of Disclosure

Concerns about the possible consequences of disclosure of mental health problems during the screening process was expressed by many participants. These concerns were, for some, characterised as fear of the unknown. Women often did not know what the implications of screening might be and, so, took a guarded approach to the process. They were sometimes cynical regarding healthcare professionals’ motivations for screening i.e., it being only about assessing risk of harm and ruling this out rather than screening having the function of offering genuine support.

*“The only question that she [health visitor] was more worried about is*,* would I self-harm or hurt the baby. I went “no”. That’s all she was more worried about*,* not dealing with the fact that*,* why am I upset?”* (Darwin et al., 2016).

Women expressed fear about loss of agency in terms of potential referrals/treatment for mental health problems. The most deep-seated and serious fear was that of child protective service involvement and even having their child removed from their care. Such fears were often linked to the anticipated consequences of disclosing thoughts of self-harm or suicide during screening.

*“I can imagine somebody may be feeling all these symptoms quite severely but how dare I say that for fear of you might take my baby off me… whether people would be honest”* (Evans et al., 2017).

Additionally, it was felt by some participants that the responses they provided, particularly via screening tools, might be misinterpreted, leading to them being labelled as being ‘mentally ill’ and being under some sort of increased surveillance. Concerns about surveillance were particularly pertinent when exploring the use of remote, self-report screening methods where there was no immediate opportunity to fully explain responses to screening questions.

### Main Theme 3: Context and Constraints

This theme describes the mediating factors that determined how women perceived screening or assessment. This is organised into the constructs of ‘Knowledge and expectations of screening’, ‘Screening methods’, and ‘Screening environment’.

#### Knowledge and Expectations of Screening

Women’s prior expectations and knowledge of mental health screening in the perinatal period was a key factor in how they perceived their experiences. Many of the studies reported that participants explained that they had not been adequately prepared for screening, to the extent that they had been quite taken aback by the questions being asked.

*“I think they could have told me what they were going to ask before I even arrived for my appointment. I had no idea that was what was coming.”* (Rollans et al., 2013).

For many, this meant that screening was unacceptable and as a result they felt unable to fully engage. The expectation existed that mental health wasn’t the remit of maternity services or, in the case of public health nurses, that they were there only to monitor the health and wellbeing of the baby. This misconception could lead to women being wary of the motivations of some professionals, that perhaps they were only interested in demonstrating that they had carried out risk management procedures at a superficial level.

*“I said I had a history of anxiety… rather than like ‘are you ok now? Are you feeling anxious?’… it was more like*,* ‘check she’s not anxious now’ and then they [midwives] were like ‘make sure you write that down*,* make sure you write down that she’s not anxious right now’…I felt a bit more like it was not actually monitoring whether I was anxious*,* it was more sort of making sure that it was recorded that I wasn’t anxious at that point*.***”*** (Yapp et al., 2019).

Women expressed an explicit need to be informed in advance about the process and to understand the purpose of screening, the nature of any questions, and how the information would be used. Furthermore, they needed reassurance that screening was a universal offering and that it was for their benefit. In the absence of such knowledge, it seemed highly probable that women would view screening as having little value and even as a potential threat, fearing the consequences of disclosing poor mental health.

*“When I booked in*,* if there could even be like*,* “These are the things we’re going to talk about*,* and it’s a really safe space and this is why you could have your partner or not have your partner.”* (Kohlhoff et al., 2021).

Whilst in some situations, women who expressed a desire to seek support with their mental health felt that screening facilitated gatekeeping to services, others viewed screening questions as being similar to a ‘test’ you either pass or fail. In this respect, because of a lack of information, women were overly concerned with conforming to a perceived expectation of them, as opposed to having their own expectations of mental health support and these being met by services.

*“I was told this was a questionnaire to identify people having problems with postnatal depression and that was it*,* there was no treatment or no consequences discussed. It wasn’t clear to me what would happen if I ticked the bad boxes. I should have been answering it for my own good*,* and people were trying to help me*,* but I wanted to get the answers right.”* (Shakespeare et al., 2003).

#### Screening Methods

This concept relates to participants’ views of the different modes, approaches, and tools used in mental health screening and how these influenced women’s perceptions of the overall screening process. Screening tools included depression questionnaires, anxiety questionnaires, clinical interviews, as well as specific depression case-finding questions. Women had contrasting views about the utility of questionnaires that required binary responses (e.g., Yes/No). Some saw completion of these as an opportunity to provide a clear indication about how they were feeling and to open up conversations, as described in the ‘opportunities’ theme. Conversely others felt that they inhibited disclosure as they represented a ‘tick-box’ exercise only, with no opportunity for further exploration. Furthermore, the use of single screening tools with a narrow focus often created the impression that the only mental health problem of interest was postnatal depression. This was, therefore, a constraining factor, with women feeling that other forms of distress, such as, for example, birth trauma or anger, were ignored or de-prioritised.

*‘‘It’s almost like once you’ve said ‘‘No I don’t’’ [feel depressed]*,* then that’s it*,* I’ve ticked that box*,* she’s not depressed she’s not going to go home and throw herself off the roof or anything so that’s that sorted’’.* (Coates et al., 2015)

Participants varied in their preferences for how they wanted screening to be conducted. Although women overwhelmingly expressed a desire to have open conversations about mental health, having an opportunity to indicate any difficulties discreetly i.e., by self-report using remote technology, such as a mobile phone-based app, or completion of a self-administered screening tool, was viewed as helpful for some. Having to answer screening questions in the moment, in front of a healthcare professional could feel uncomfortable due to embarrassment and feeling under pressure. These participants expressed a preference for space and time to consider their responses which also helped them to feel less judged.

*“It felt better knowing that*,* on paper*,* I wasn’t been judged and could get help if I needed.”* (Mann et al., 2015).

The type of language used (whether written or spoken) influenced the extent to which women felt positive or negative about screening. Certain questions were described as being too blunt and medicalised and others were criticised for having stigmatising connotations, therefore inhibiting disclosure. The wording of questions that probed suicide or self-harm, were viewed as especially sensitive in nature and, as such, elicited strong views. Participants disliked some of the phrases used to describe suicidal ideation, expressing that they might be deemed offensive or inappropriate. Some participants objected to the concept of being asked about suicide or self-harm at all.

*“Many people think that during the time when you’re antenatal you should not talk about suicidal thoughts…it has detrimental effects on the baby also*,* to talk about it.” *(Fellmeth et al., 2022).

An additional factor leading to difficulties with screening was whether women understood what the questions were asking and whether they felt the available responses adequately captured their experiences. For example, being unsure whether to mark ‘yes’ in answer to a question when considering the severity or fluctuation of their symptoms. Again, this was often most marked in reference to suicide/self-harm questions, which participants often found confusing or ambiguous. Similarly, women often felt that the reference timeframe used in some screening tools was too restrictive, for example, being asked to respond based on only the past seven days. This prevented them from feeling able to disclose mental health difficulties that occurred less recently than was being asked about. Consistently, participants conveyed the importance of context and time in terms of disclosing their mental health problems, and methods/approaches that did not allow for this might be met with frustration. Women did not want to be seen only through the narrow lens of their pregnancy/postnatal experience.

*“You talk about things because they are important to talk about not cause they happened one week ago! It is a real whitefella way to start. It’s like you’re in or you’re out. You see that hey? Like what happens if it was a bit longer*,* then the lady might think oh no*,* it’s not important I won’t talk about that”.* (Carlin et al., 2019)

#### Screening Environment

The environment in which screening took place was instrumental in determining the nature of participants’ experiences. The need for privacy was most central to this concern. The location, whether at home or in a clinical setting, was a matter of personal preference and some women preferred to have a support person, such as a partner, with them whereas others did not find a support person helpful. When women felt adequately prepared for screening, they were able to decide whether the presence of another person would be desirable, ensuring the required privacy.

*“That first Edinburgh test*,* to have it filled in and then talked about in front of everybody else was just terrible.”* (Shakespeare et al., 2003).

Women were acutely aware when healthcare professionals were overloaded with tasks and were unable to dedicate enough time and attention to screening. The effect of this was that care could feel extremely mechanistic and depersonalised, which impacted women’s screening experiences negatively as they needed the encounter to feel relaxed and unhurried.

*“There were a lot of questions and that was just part of it… it felt like an appointment where certain things had to be done you know… I don’t feel like there was… room…”* (Yapp et al., 2019).

Women wanted to feel that the environment was a safe space and their impression of, and connection with, the person conducting the screening was key to this. A positive relationship that engendered trust was important and this could happen more easily when healthcare professionals demonstrated kindness, empathy, and genuine interest and care regarding women’s responses to questions about mental health. It was also important that the person screening appeared to be comfortable themselves and they projected an impression of competence.

*“I think just their general demeanour like how they are*,* how they speak to you*,* the tone of their voice*,* their understanding of being pregnant and kind of what*,* all of those emotional things.”* (Williams et al., 2016).

Some women reported that receiving continuity of care from a single healthcare professional helped them to build this type of trusting relationship, yet others suggested that the persons’ interpersonal skills were more important than familiarity. For women from minoritised communities, anonymity and confidentiality was assured when the person screening was someone from outside of their community and they were able to utilise a private space.

*“Actually*,* it’s better to be in a place that’s. . face-to-face interview with one person. When they asked me*,* about how’s your relationship with your husband*,* what are you feeling*,* then there are around me other Afghans women*,* and they are. . they were looking at me*,*. . what’s my answer for this question.”* (Willey et al., 2020).

Nevertheless, others reported feeling that speaking with, or being supported by, someone from their own community who really understood their unique challenges would help them to feel more are ease and understood. This was particularly true for women from communities where there was a general mistrust of services due to intergenerational trauma and historical and current structural racism. Women felt that healthcare professionals made assumptions about their preferences and needs based on stereotyping.

*“They [midwives] probably assumed I had support from whānau*,* but no one actually asked… I think they just expect that whānau will handle it*,* so they don’t bother to ask how you’re coping”* (Hayward et al., 2025).

## Discussion

This meta-ethnographic review is the first to examine the *process* of mental health screening and assessment in the perinatal period, thus addressing a demonstrable need to more adequately understand how and why women do and do not find screening acceptable. Using a line of argument synthesis based on an analysis of 38 papers, we were able to identify three over-arching aspects of the screening process that influenced and determined how women perceived it. These were: (i) Knowledge and expectations of screening, (ii) The screening method, and (iii) The screening environment. Participants reported that screening presented positive opportunities in several ways: by providing an outlet for emotional expression and a way to communicate important information; by having positive effects, for example providing insight and acceptance, and in acting as an intervention where participants were motivated to seek help and support. Nevertheless, screening was also viewed as a difficult experience. This occurred when women were unable to see any real value in screening, when they felt the impact of stigma and perceived judgment, and when they feared the implications of screening and potential consequences of any disclosure.

Understandably, in common with the findings of Brealey et al. (2010) it was clear that women needed, first and foremost, to feel fully informed about mental health screening and to feel safe and relaxed in the screening environment. However, by focusing our analysis on the screening process we were able to probe further and to provide a more elaborate, nuanced and comprehensive insight into women’s perceptions and reflections. This advances understanding of how and why screening acceptability/unacceptability occurs and has important implications for health care practice. As such, attention is drawn to the following three novel findings.

The first key and novel finding was that women’s preferences for how they would like to be asked about their mental health during the screening process varied widely. Both Brealey et al. (2010) and El-Den et al. (2015) argue screening setting is an important factor influencing acceptability, with home screening being seen as preferable. Contrastingly, we found that women were not unanimous in a preference for home as a screening setting, nor was the setting the most salient factor in women’s accounts. Three putative reasons may underlie this difference. First, the reviews by Brealey et al. and El-Den et al. focused only on screening for postnatal depression and primarily using the EPDS, whereas the current review included screening for any mental health problem across the perinatal period using any instrument. This meant that the studies included were rather more heterogeneous in that they were conducted across a range of countries; within marginalised groups; for different screening purposes; and with screening methods, including the use of digital screening with mobile apps and tablets (Doherty et al., 2018; Dyurich & Oliver, 2020; Willey et al., 2020; Witcraft et al., 2025). Participants samples included in our review had disparate life circumstance and needs, ranging from participants recruited from private maternity hospitals in Australia (Kohlkoff et al., 2021) to participants living with HIV in Kenya (Velloza et al., 2020). Second, and relatedly, since 2010 expectations may have been altered because home visits as part of routine postnatal care have been reduced overall, particularly since the Covid-19 pandemic, which saw a shift towards offering more virtual and clinic based care (Cross-Sudworth et al., 2024). There is emerging evidence for the acceptability of tele mental health services for perinatal women, for example, when accessing psychiatry care (Ackerman et al., 2021), and the review by Clarke et al. (2024) found that women were generally accepting of digital screening methods. Our findings also support this view, but take them further because we found that the potential opportunities and difficulties presented by screening were not contingent on any particular method or mode, but were affected by specific aspects of the chosen screening method, for example, question focus, response options, language, as well as other contextual and constraining factors such as the need for a private space, which was one of the perceived benefits of digital screening (Dyurich & Oliver, 2020; Willey et al., 2020; Witcraft et al., 2025). Finally, it is important to be cognisant that home is not always a safe space for all women, with a recent meta-analysis reporting worldwide prevalence of intimate partner violence (IPV) in pregnancy being as high as 25% (Román-Gálvez et al., 2021). In addition, significant associations have been reported between perinatal mental health problems and having experienced IPV (Howard et al., 2013; Paulson, 2022). Our findings reveal the importance of the screening environment feeling like a safe space and so it follows that women experiencing abuse would prefer for conversations about their mental health to take place outside the home.

The second key and novel finding of this review was that various aspects of the screening process resulted in women seeing little value in it. This manifested in them expressing ambivalence or scepticism and ultimately disengaging with screening. The literature on help-seeking for perinatal psychological distress has revealed some of the reasons that women may choose not to disclose or seek help for possible mental health problems. These include knowledge about perinatal mental health and the roles of healthcare professionals and the help available, as well as an inability to recognise mental health problems (Button et al., 2017; Hadfield & Wittkowski, 2017; Rouhi et al., 2019; Webb et al., 2023). These intrinsically located factors resonate with the findings of the current review. However, our analysis shifts the focus by re-framing these factors as failures in the screening process itself i.e., aspects such as a lack of provision of adequate information, restrictive and unhelpful screening questions/response options and non-facilitative environments. For instance, we have highlighted that an important opportunity presented by screening is that it can assist women with recognition of mental health problems. However, this potential impactful opportunity was lost when questions were either confusing or failed to capture what women were feeling. Screening questions were often perceived as being too narrow both in their focus and in the available response options. This finding lends support to the call for a broader understanding of perinatal mental health problems in terms of screening, treatment, and within the discourse of public agencies (Dossett et al., 2024). A pertinent example is a recent meta-synthesis which found that loneliness is often central in experiences of perinatal depression (Adlington et al., 2023), yet screening tools may not probe this construct, meaning a potentially significant indicator of postnatal depression could be missed. Furthermore, participants reported difficulties answering questions in the context of being pregnant/postnatal, particularly when those questions had been developed for more general mental health settings. This, perhaps, explains why participants have sometimes reported preferring a pregnancy-specific questionnaire over general anxiety assessments (Evans et al., 2017), and the need for more anxiety measures to be developed and validated in antenatal populations has been recently recognised (Rondung et al., 2024). Such findings consolidate the call for attention to be focused on validating and amending existing tools so that they can be used to identify mental health problems across countries, cultures, and socioeconomic groups, and at particular ‘high risk’ times during the perinatal period (Sambrook Smith et al., 2022). Moreover, our analysis revealed that women can feel frustrated and constrained by screening if there is no ‘space’ for conversations that enable them to explain and fully describe the nature of their distress, leading to women feeling ignored and let down, reinforcing the view that maternity healthcare professionals are not appropriately trained or placed to meet women’s mental health needs This aligns with the findings of a recent review, in which support from both healthcare professionals and women for conversational psychosocial assessment tools was reported (Gram et al., 2024). All of these factors may converge to negatively influence women’s perceptions of screening. This means that even when women are initially open to talking about mental health and intend to disclose their distress, the actual process can alter that intention entirely, diminishing any potential value. Nonetheless, some participants expressed feeling that nothing could be done about their mental health concerns, or that the nature of the changes that happen in the perinatal period mean that distress is expected and/or inevitable. For these women, there was a kind of acceptance of their mental health struggles and as a consequence they placed little value on the screening process.

A final, important novel finding of the current review pertains to screening for suicidal experiences, such as those probing thoughts of self-harm and thoughts of death/dying by suicide. These questions were generally found to be the most problematic to participants across the studies and women expressed that a person would have to be feeling very desperate and in need of urgent help to ‘admit to’ such feelings. This is a concerning view, as there is an imperative to identify any type of suicidal thoughts regardless of severity, when considering that suicidal ideation is the most important risk factor for suicide attempts or death by suicide in the perinatal period (Orsolini et al., 2016).The difficulties highlighted under the construct of ‘internalised stigma and shame’ most often related to being asked about suicide and self-harm, which is unsurprising considering the persistent and ingrained stigmatisation of suicide and self-harm in general (Carpiniello & Pinna, 2017), but particularly when paired with motherhood (Biggs et al., 2023). Similar thoughts and feelings about the stigma that accompanies suicidal thoughts and acts have been documented in reviews exploring the experiences of mothers with severe mental health problems (Dolman et al., 2013; Harries et al., 2023). Some participants in the current review revealed that one source of internalised stigma, shame, and lack of acceptance which surfaced alongside the possibility of being asked about suicide or self-harm was due to the unacceptability of such an ‘admission’ within their communities or religion (Fellmeth et al., 2022; Velloza et al., 2020). For these women, there is a cumulative multi-layering effect of the stigma associated with suicidal feelings combined with maternal status, amplified by a perceived cultural and/or religious judgment. This fear of disclosure alongside the racial and ethnic disparities in perinatal mental health that exist, such as the higher rates of suicidal ideation, behaviour and deaths by suicide identified amongst black women (Salihu et al., 2022; Tabb et al., 2023), perhaps, exemplifies a reciprocal relationship, whereby each feeds into the other. Coupled with this, women from minority ethnic groups are actually less likely than their non-minority counterparts to be screened/have mental health problems identified (Prady et al., 2021; Sidebottom et al., 2021). In the only study in this review to examine suicide screening specifically (Dudeney et al., 2024a), the type of language that women judged as appropriate to ask about suicidal experiences was highly variable and there was no consensus about phrasing that was more or less acceptable. However, participants across studies found difficulties with the ambiguous nature of some of the suicide questions. This was either because the language was not clear and direct enough (Dudeney et al., 2024a) or because they struggled to reconcile what they were experiencing with the description presented to them by the question (Poole et al., 2006). Similar findings have been reported in non-perinatal populations personally affected by suicide regarding different preferences for the language used to describe suicidal behaviour (Padmanathan et al., 2019). Taken together, these findings point to an urgent imperative to develop culturally sensitive ways of enquiring about suicidal thoughts, feelings, urges, plans, images and behaviours, in conjunction with the development of questions and measures that women feel are representative of their own experiences and that are acceptable to them.

### Strengths and Limitations

There were three main strengths of the current review. First, this is the first review to document and focus specifically on women’s views and experiences of the *process* of perinatal mental health screening, synthesising literature that investigated a range of screening approaches using a variety of qualitative methods. Second, we utilised a systematic and comprehensive searching approach in combination with purposive sampling. This ensured that we were able to maintain a broad and inclusive scope i.e., timescales, populations and methods, yet conduct an interpretative qualitative analysis that remained close to the original data. Third, the final included studies were rated as of high and medium quality by two individual screeners and included the voices of women from diverse study samples. Nevertheless, findings should be considered with certain limitations in mind. Although quality appraisal of the studies was rated high/medium across the board, only 36% of studies demonstrated adequate reflexivity in terms of considering the relationship between the researcher and participants, raising questions about how observer bias may have been navigated. Similarly, some of the included studies did not report the ethnicity or nationality of their participants or reported imprecise data e.g. ‘White’ or ‘Other’. This means that (other than the studies where participants were purposefully sampled due to their ethnic, cultural or religious status) we cannot draw conclusions about any intersecting needs or difficulties women may have been experiencing in relation to screening. This is especially important as there is now increasing evidence of the urgent need for tailored screening programmes to meet the needs of migrant women in the perinatal period (Verschuuren et al., 2025).

## Conclusion

This meta-ethnographical review of the literature regarding women’s views and experiences of perinatal mental health screening, presents a lines of argument synthesis which identified important contextual and constraining factors that impact women’s perceptions of the process. Novel findings revealed that mental health screening in the perinatal period is not simply a passive process, in that it presents an unmissable opportunity to act as a positive intervention in multiple ways. These include opportunities for meeting public health promotion goals by aiding personal insight and motivation for help-seeking and for progress in addressing the urgent needs of suicidal women in the perinatal period. For this to occur, those involved in planning and implementing screening programmes need to pay attention to all aspects of the process that could act as inhibiting factors and, therefore, negatively impact the perceived value of screening. Offering flexibility and choice with methods and modes of screening, in enabling environments that are suited to the diverse needs of women is key. Future research might benefit from a focus on understanding the screening needs of minoritised communities as well as effective approaches to screening for suicidal experiences.

## Supplementary Information

Below is the link to the electronic supplementary material.

Supplementary Material 1

Supplementary Material 2

Supplementary Material 3

Supplementary Material 4

Supplementary Material 5

## Data Availability

No datasets were generated or analysed during the current study.
