# Supplementary material for: Women’s Experiences of Screening and Assessment of Mental Health Problems in the Perinatal Period: A Systematic Review and Meta-synthesis
Source: Clin Child Fam Psychol Rev. 2026 May 8;29(3):506–34. doi: 10.1007/s10567-026-00571-9 (PMC13562364; doi:10.1007/s10567-026-00571-9)
Supplement: Supplementary file 1 [file 10567_2026_571_MOESM1_ESM.docx]

| 1. | perinatal.mp. or Perinatal Care/ |  |  |  |  |
| --- | --- | --- | --- | --- | --- |
| 2. | Prenatal Care/ or antenatal.mp. or Pregnancy/ |  |  |  |  |
| 3. | postnatal.mp. or Postnatal Care/ |  |  |  |  |
| 4. | 1 or 2 or 3 |  |  |  |  |
| 5. | assess*.mp. |  |  |  |  |
| 6. | screen*.mp. |  |  |  |  |
| 7. | Disclosure/ or disclos*.mp. |  |  |  |  |
| 8. | 5 or 6 or 7 |  |  |  |  |
| 9. | Mental Health/ |  |  |  |  |
| 10. | mental illness.mp. or Mental Disorders/ |  |  |  |  |
| 11. | Anxiety/ |  |  |  |  |
| 12. | exp Depression, Postpartum/ |  |  |  |  |
| 13. | Suicidal Ideation/ or Suicide/ or suicid*.mp. or Self-Injurious Behavior/ |  |  |  |  |
| 14. | 9 or 10 or 11 or 12 or 13 |  |  |  |  |
| 15. | Qualitative Research/ |  |  |  |  |
| 16. | interview*.mp. |  |  |  |  |
| 17. | "women's experiences".mp. |  |  |  |  |
| 18. | 15 or 16 or 17 |  |  |  |  |
| 19. | 4 and 8 and 14 and 1 |  |  |  |  |

**Medline**

**Embase**

| 1. | perinatal period/ or perinatal care/ or perinatal.mp. |  |  |  |  |
| --- | --- | --- | --- | --- | --- |
| 2. | pregnancy.mp. or pregnancy/ |  |  |  |  |
| 3. | antenatal.mp. or prenatal care/ |  |  |  |  |
| 4. | postnatal care/ or postnatal.mp. |  |  |  |  |
| 5. | 1 or 2 or 3 or 4 |  |  |  |  |
| 6. | assess*.mp. |  |  |  |  |
| 7. | screen*.mp. |  |  |  |  |
| 8. | disclos*.mp. or interpersonal communication/ |  |  |  |  |
| 9. | 6 or 7 or 8 |  |  |  |  |
| 10. | mental health.mp. or mental health/ |  |  |  |  |
| 11. | mental illness.mp. or mental disease/ |  |  |  |  |
| 12. | puerperal depression/ or postnatal depression/ |  |  |  |  |
| 13. | anxiety disorder/ or anxiety/ or anxiety assessment/ or anxiety.mp. |  |  |  |  |
| 14. | suicidal ideation/ or suicide/ or suicid*.mp. or suicidal behavior/ |  |  |  |  |
| 15. | 10 or 11 or 12 or 13 or 14 |  |  |  |  |
| 16. | qualitative research/ or qualitative analysis/ or qualitative.mp. |  |  |  |  |
| 17. | interviews.mp. or interview/ |  |  |  |  |
| 18. | 16 or 17 |  |  |  |  |
| 19. | 5 and 9 and 15 and 18 |  |  |  |  |
| 20. | limit 19 to journal |  |  |  |  |
| 21. | limit 20 to article |  |  |  |  |

**Midirs**

| 1. | perinatal.mp. [mp=abstract, heading word, title] |  |  |  |  |
| --- | --- | --- | --- | --- | --- |
| 2. | pregnancy.mp. [mp=abstract, heading word, title] |  |  |  |  |
| 3. | postnatal.mp. [mp=abstract, heading word, title] |  |  |  |  |
| 4. | antenatal.mp. [mp=abstract, heading word, title] |  |  |  |  |
| 5. | 1 or 2 or 3 or 4 |  |  |  |  |
| 6. | assess*.mp. [mp=abstract, heading word, title] |  |  |  |  |
| 7. | screen*.mp. [mp=abstract, heading word, title] |  |  |  |  |
| 8. | disclos*.mp. [mp=abstract, heading word, title] |  |  |  |  |
| 9. | 6 or 7 or 8 |  |  |  |  |
| 10. | mental health.mp. [mp=abstract, heading word, title] |  |  |  |  |
| 11. | mental illness.mp. [mp=abstract, heading word, title] |  |  |  |  |
| 12. | depress*.mp. [mp=abstract, heading word, title] |  |  |  |  |
| 13. | anxiety.mp. [mp=abstract, heading word, title] |  |  |  |  |
| 14. | suicid*.mp. [mp=abstract, heading word, title] |  |  |  |  |
| 15. | self-harm.mp. [mp=abstract, heading word, title] |  |  |  |  |
| 16. | 10 or 11 or 12 or 13 or 14 or 15 |  |  |  |  |
| 17. | experience*.mp. [mp=abstract, heading word, title] |  |  |  |  |
| 18. | qualitative.mp. [mp=abstract, heading word, title] |  |  |  |  |
| 19. | interview*.mp. [mp=abstract, heading word, title] |  |  |  |  |
| 20. | 17 or 18 or 19 |  |  |  |  |
| 21. | 5 and 9 and 16 and 20 |  |  |  |  |
| 22. | limit 21 to original research |  |  |  |  |

**Cinahl Plus**

**Print Search History**

|  | Tuesday, August 08, 2023 10:55:30 AM |
| --- | --- |

| **#** | **Query** | **Limiters/Expanders** | **Last Run Via** | **Results** |
| --- | --- | --- | --- | --- |
| S20 | S15 AND S16 AND S17 AND S19 | Expanders - Apply equivalent subjects Search modes - Boolean/Phrase | Interface - EBSCOhost Research Databases Search Screen - Advanced Search Database - CINAHL Plus | Display |
| S19 | S12 OR S13 OR S14 OR S18 | Expanders - Apply equivalent subjects Search modes - Boolean/Phrase | Interface - EBSCOhost Research Databases Search Screen - Advanced Search Database - CINAHL Plus | Display |
| S18 | (MH "Appointments and Schedules") | Expanders - Apply equivalent subjects Search modes - Boolean/Phrase | Interface - EBSCOhost Research Databases Search Screen - Advanced Search Database - CINAHL Plus | Display |
| S17 | S9 OR S10 OR S11 | Expanders - Apply equivalent subjects Search modes - Boolean/Phrase | Interface - EBSCOhost Research Databases Search Screen - Advanced Search Database - CINAHL Plus | Display |
| S16 | S5 OR S6 OR S7 OR S8 | Expanders - Apply equivalent subjects Search modes - Boolean/Phrase | Interface - EBSCOhost Research Databases Search Screen - Advanced Search Database - CINAHL Plus | Display |
| S15 | S1 OR S2 OR S3 OR S4 | Expanders - Apply equivalent subjects Search modes - Boolean/Phrase | Interface - EBSCOhost Research Databases Search Screen - Advanced Search Database - CINAHL Plus | Display |
| S14 | (MH "Edinburgh Postnatal Depression Scale") OR "edinburgh postnatal depression scale" OR (MH "Self-Rating Depression Scale") OR (MH "Scales") | Expanders - Apply equivalent subjects Search modes - Boolean/Phrase | Interface - EBSCOhost Research Databases Search Screen - Advanced Search Database - CINAHL Plus | Display |
| S13 | screen* | Expanders - Apply equivalent subjects Search modes - Boolean/Phrase | Interface - EBSCOhost Research Databases Search Screen - Advanced Search Database - CINAHL Plus | Display |
| S12 | (MH "Diagnosis, Psychosocial") OR "psychosocial assessment" | Expanders - Apply equivalent subjects Search modes - Boolean/Phrase | Interface - EBSCOhost Research Databases Search Screen - Advanced Search Database - CINAHL Plus | Display |
| S11 | (MH "Interviews") OR (MH "Semi-Structured Interview") OR (MH "Unstructured Interview") | Expanders - Apply equivalent subjects Search modes - Boolean/Phrase | Interface - EBSCOhost Research Databases Search Screen - Advanced Search Database - CINAHL Plus | Display |
| S10 | "women's experiences" | Expanders - Apply equivalent subjects Search modes - Boolean/Phrase | Interface - EBSCOhost Research Databases Search Screen - Advanced Search Database - CINAHL Plus | Display |
| S9 | (MH "Qualitative Studies") OR "qualitative" | Expanders - Apply equivalent subjects Search modes - Boolean/Phrase | Interface - EBSCOhost Research Databases Search Screen - Advanced Search Database - CINAHL Plus | Display |
| S8 | (MH "Postpartum Psychosis") OR "postpartum psychosis" | Expanders - Apply equivalent subjects Search modes - Boolean/Phrase | Interface - EBSCOhost Research Databases Search Screen - Advanced Search Database - CINAHL Plus | Display |
| S7 | (MH "Anxiety") OR "anxiety" | Expanders - Apply equivalent subjects Search modes - Boolean/Phrase | Interface - EBSCOhost Research Databases Search Screen - Advanced Search Database - CINAHL Plus | Display |
| S6 | (MH "Depression") OR (MH "Bipolar Disorder") OR (MH "Depression, Postpartum") OR "depress*" | Expanders - Apply equivalent subjects Search modes - Boolean/Phrase | Interface - EBSCOhost Research Databases Search Screen - Advanced Search Database - CINAHL Plus | Display |
| S5 | (MH "Mental Disorders") OR "perinatal mental health or perinatal mental illness or maternal mental health" | Expanders - Apply equivalent subjects Search modes - Boolean/Phrase | Interface - EBSCOhost Research Databases Search Screen - Advanced Search Database - CINAHL Plus | Display |
| S4 | (MH "Postnatal Period") OR (MH "Postnatal Care") OR "postnatal" | Expanders - Apply equivalent subjects Search modes - Boolean/Phrase | Interface - EBSCOhost Research Databases Search Screen - Advanced Search Database - CINAHL Plus | Display |
| S3 | (MH "Pregnancy") | Expanders - Apply equivalent subjects Search modes - Boolean/Phrase | Interface - EBSCOhost Research Databases Search Screen - Advanced Search Database - CINAHL Plus | Display |
| S2 | (MH "Prenatal Care") OR "antenatal" | Expanders - Apply equivalent subjects Search modes - Boolean/Phrase | Interface - EBSCOhost Research Databases Search Screen - Advanced Search Database - CINAHL Plus | Display |
| S1 | (MH "Perinatal Period") OR (MH "Perinatal Care") OR "perinatal" | Expanders - Apply equivalent subjects Search modes - Boolean/Phrase | Interface - EBSCOhost Research Databases Search Screen - Advanced Search Database - CINAHL Plus | Display |

Bottom of Form

**Web of Science**

**Web of Science**

**perinatal OR pregnancy OR antenatal OR postnatal (Topic) and "postpartum depression" OR "postnatal depression" OR "mental health" OR "perinatal mental illness" OR suicid* (Topic) and screen* OR assess* OR disclos* (Topic) and qualitative OR interviews OR women's experiences (Topic)**

**Title:** Women’s Experiences of Screening and Assessment of Mental Health Problems in The Perinatal Period: A Systematic Review and Meta-synthesis

**Journal:** Clinical Child and Family Psychology Review

**Authors:** Kerry Hozhabrafkan, Patricia Gooding, Anja Wittkowski, Sanaa Hyder and Sarah Peters

**Affiliation:** University of Manchester

**Corresponding Author Email:** Kerry.hozhabrafkan@postgrad.manchester.ac.uk
