# Supplementary material for: Women’s Experiences of Screening and Assessment of Mental Health Problems in the Perinatal Period: A Systematic Review and Meta-synthesis
Source: Clin Child Fam Psychol Rev. 2026 May 8;29(3):506–34. doi: 10.1007/s10567-026-00571-9 (PMC13562364; doi:10.1007/s10567-026-00571-9)
Supplement: Supplementary file 2 [file 10567_2026_571_MOESM2_ESM.docx]

**Table S1**

*Data Richness Scale Used to Guide Purposive Sampling Decisions*

| **Score** | **Measure** | **Example** |
| --- | --- | --- |
| 1 | Very little qualitative data presented that relate to the synthesis objective. Those findings that are presented are fairly descriptive. | For example, a mixed methods study using open ended survey questions or a more detailed qualitative study where only part of the data relates to the synthesis objective |
| 2 | Some qualitative data presented that relate to the synthesis objective | For example, a limited number of qualitative findings from a mixed methods or qualitative study |
| 3 | A reasonable amount of qualitative data that relate to the synthesis objective | For example, a typical qualitative research article in a journal with a smaller word limit and often using simple thematic analysis |
| 4 | A good amount and depth of qualitative data that relate to the synthesis objective | For example, a qualitative research article in a journal with a larger word count that includes more context and setting descriptions and a more in-depth presentation of the findings |
| 5 | A large amount and depth of qualitative data that relate in depth to the synthesis objective. | For example, from a detailed ethnography or a published qualitative article with the same objectives as the synthesis |

*Reproduced from:* Ames, H., Glenton, C., & Lewin, S. (2019). Purposive sampling in a qualitative evidence synthesis: a worked example from a synthesis on parental perceptions of vaccination communication. *BMC Medical Research Methodology*, *19*(1). <https://doi.org/10.1186/s12874-019-0665-4>

**Title:** Women’s Experiences of Screening and Assessment of Mental Health Problems in The Perinatal Period: A Systematic Review and Meta-synthesis

**Journal:** Clinical Child and Family Psychology Review

**Authors:** Kerry Hozhabrafkan, Patricia Gooding, Anja Wittkowski, Sanaa Hyder and Sarah Peters

**Affiliation:** University of Manchester

**Corresponding Author Email:** Kerry.hozhabrafkan@postgrad.manchester.ac.uk
