# Supplementary material for: Women’s Experiences of Screening and Assessment of Mental Health Problems in the Perinatal Period: A Systematic Review and Meta-synthesis
Source: Clin Child Fam Psychol Rev. 2026 May 8;29(3):506–34. doi: 10.1007/s10567-026-00571-9 (PMC13562364; doi:10.1007/s10567-026-00571-9)
Supplement: Supplementary file 3 [file 10567_2026_571_MOESM3_ESM.docx]

**Table S2**

*Summary of the Seven Phases of Meta-ethnography*

| Phase | Description |
| --- | --- |
| **1. Getting started** | Identifying the issue of interest and deciding whether a synthesis is needed and the suitability of meta-ethnography as a medium though which to explore the issue |
| **2. Deciding what is relevant** | Identifying and selecting studies to synthesise |
| **3. Reading the studies** | Repeated reading of the studies, familiarising with and collating key concepts/metaphors/themes |
| **4. Determining how the studies are related** | Determining the relationship between the studies to be synthesized i.e. comparing key concepts/metaphors/themes and identifying similarities or differences to decide what type of translation is indicated e.g. reciprocal/refutational |
| **5. Translating the studies into one another** | Comparing key concepts within and across the studies to check meaning and organising them to construct new interpretations, expressed as third order constructs |
| **6. Synthesising translations** | Described as ‘making the whole into something more than the parts alone imply’. Creating an over-arching conceptual interpretation of the new third order constructs in the form of a line of argument synthesis |
| 7. Expressing the synthesis | Communicating the synthesis to the target audience |

**Title:** Women’s Experiences of Screening and Assessment of Mental Health Problems in The Perinatal Period: A Systematic Review and Meta-synthesis

**Journal:** Clinical Child and Family Psychology Review

**Authors:** Kerry Hozhabrafkan, Patricia Gooding, Anja Wittkowski, Sanaa Hyder and Sarah Peters

**Affiliation:** University of Manchester

**Corresponding Author Email:** Kerry.hozhabrafkan@postgrad.manchester.ac.uk
