# Supplementary material for: Women’s Experiences of Screening and Assessment of Mental Health Problems in the Perinatal Period: A Systematic Review and Meta-synthesis
Source: Clin Child Fam Psychol Rev. 2026 May 8;29(3):506–34. doi: 10.1007/s10567-026-00571-9 (PMC13562364; doi:10.1007/s10567-026-00571-9)
Supplement: Supplementary file 4 [file 10567_2026_571_MOESM4_ESM.docx]

**Table S3**

*Studies Excluded using Purposive Sampling*

| **Authors & Year** | **Participants** | **Setting** | **Study aims** | **Data richness rating** | **Comments** |
| --- | --- | --- | --- | --- | --- |
| Clark (2000) | Antenatal women | England | To examine the use of the EPDS by health visitors to detect low mood in pregnancy | 2 | Participants included health visitors as well as women so amount of relevant data presented is limited |
| Matthey et al (2005) | Antenatal and postnatal women (English speaking & non-English speaking | Australia | To examine acceptability of routine antenatal psychosocial assessment | 2 | There is some data presented relating to answering EPDS questions but many of the findings relate to the wider psychosocial assessment questions |
| Leigh & Milgrom (2007) | Antenatal women | Australia | To examine acceptability of screening for antenatal depression in routine antenatal care | 2 | Telephone interviews. Very little qualitative data presented relating to women’s views specifically about answering the questions, there is more about feedback received from midwives |
| Slade et al (2010) | Postnatal women | UK | To explore postnatal women’s experiences of identification and management of symptoms of depression | 2 | A small amount of data presented relating specifically to the experience of being screened. Most of the findings are about perceptions of the health visitor/their role, help seeking and the experience of the intervention/support |
| Stapleton et al (2013) | Antenatal women with refugee status | Australia | To explore cross-cultural application of the EPDS and any difficulties with administration to women from refugee backgrounds | 1 | Findings report comprehension and acceptability of items on the EPDS via interpreters employed as RAs as well as clinic staff. Although service users participated in focus groups and free text surveys, the data is not quoted directly from the women. |
| Phillips & Thomas (2015) | Antenatal and perinatal women with pre-existing diagnosis of mental illness | UK | To explore the expectations and experiences of the first antenatal booking appointment of women with pre-existing mental illness diagnosis | 2 | The majority of the data presented relates to experiences more broadly e.g. motherhood, feelings about disclosure and expectations of ongoing care. Data specifically about the process of being asked about their mental health is limited. |
| Brugha et al (2016) | Antenatal women | UK | To assess feasibility and acceptability to pregnant women ‘not at risk’ of depression and to carers and midwives of training of community midwives in psychological approaches to prevent the development of depression in pregnancy | 2 | There is some data relating specifically to women’s views about midwives administering the EPDS, but this is limited to a couple or points with quotes. The rest of the findings are about experiences of receiving support from the midwife as well as midwives perspectives |
| Doering et al (2017) | Postnatal women | USA | To describe the needs and challenges of home visitors, their supervisors and clients when conducting depression screening | 2 | Most of the participant data (from focus groups) relates to the professional groups. Only four quotes from postnatal women about being screened using the EPDS |
| Marley et al (2017) | Antenatal & Postnatal women  Aboriginal women | Australia | To determine if the KMMS is a valid and acceptable method of identifying Kimberley Aboriginal perinatal women at risk of anxiety or depressive disorders compared to a semi-structured clinical interview. | 1 | A mixed methods study. The section on acceptability does provide some good insight into women’s views, but they primarily refer to completing the psychosocial aspects of the KMMS as opposed the mental health screening specifically |
| Johnsen et al (2018) | Antenatal women | Denmark | To explore women’s experiences of self-reporting their health prior to the first antenatal visit | 2 | The findings that clearly relate to mental health screening specifically (rather than to physical health or general pregnancy concerns) are limited |
| Gannan et al (2019) | Postnatal women who were immigrants | Canada | To explore factors that contribute to PPD and health service accessibility, and the role of health services in supporting immigrant women with PPD. | 1 | Most of the data relates to the women’s experience of services more generally. Only a very small snippet described answering questions about mental health |
| Oh et al (2020) | Antenatal and postnatal women with self-reported anxiety | UK | To explore women’s experiences of the identification and management of PNA and their engagement with healthcare professionals. |  | There is some very insightful data relating the help seeking for anxiety, however very little pertains to the screening process (one paragraph) |
| Premji et al (2021) | Antenatal and postnatal women | China | To engage multiple stakeholders (i.e., healthcare providers, pregnant and postpartum women and their families, and policymakers), to explore the acceptance, utility and readiness for a PDSM program in Anhui Province | 2 | The majority of the data comes from other stakeholder i.e. policy makers, healthcare providers and family members. There is small amount of data regarding women’s experiences of screening but these are quite descriptive |
| Tourtelot et al (2021) | Antenatal women | USA | To evaluate how providers in a busy urban practice with universal depression screening and co-located behavioural health services responded to positive screens and to explore patient expectations and attitudes towards positive screens. | 2 | Findings mostly relate to diagnosis and documentations and plans for ongoing care for mental health problems. Some data relevant to participant views about the process, but this is limited. |
| Xu et al (2021) | Postnatal women | USA | To elicit the perspectives of individuals with a traumatic birth experience on barriers and facilitators to receiving mental health support in the postpartum period. | 1 | There was a very small amount of data relating to screening, which was mostly about the lack of screening/attention to mental health |
| Yu et al (2021) | Postnatal women | USA | To assess culturally tailored strategies for the implementation of CI to evaluate the cultural appropriateness of Chinese version of the EPDS (C-EPDS) with Chinese immigrant women in the United States. | 1 | This was a cognitive interview study. The findings are heavily focused on understanding of the questions which was very specific, rather than women’s views/feelings about answering them |
| Eisner et al (2022) | Antenatal women | UK | To evaluate the feasibility and acceptability of an app for screening for depression using the EPDS | 2 | This proof of concept study reported on the usability and validity of the screening app, with a very small amount of qualitative data relating to women’s views. Extensive participant quotes are included in the supplementary information but is not supported by author analysis. It is stated that this will be reported separately |
| Kroll-desrosiers (2022) | Antenatal and postnatal women | USA | To explore Veterans’ perspectives on implementing USPSTF recommendations that would refer them to a VA mental health counsellor at the time of pregnancy confirmation, reflecting on their recent and ongoing experiences of mental healthcare and depression screening during the perinatal period. | 2 | There were some findings that were relevant to the screening experience, but these were brief and limited to three, brief points. |
| Pinar et al (2022) | Postnatal women | UK | To explore women's experiences of support and care received from maternity healthcare professionals for perinatal low mood or depression, and healthcare professionals' experiences of providing support and care for women experiencing perinatal low mood or depression | 2 | The small amount of data specifically relating to the screening process was mainly descriptive about the procedural aspects of screening e.g. what the HCP did/did not do. |
| Rice et al (2022) | Postnatal | UK | To examine women’s experiences with peer support for mental health problems during the perinatal period | 1 | A minimal amount of data regarding women’s experience of being asked about their mental health. |
| Savory et al (2022) | Antenatal | UK | To explore the experiences of women during pregnancy with mild to moderate mental health problems and describe the barriers to receiving support in relation to their mental health. | 1 | A minimal amount of data regarding women’s experience of being asked about their mental health. |

**Title:** Women’s Experiences of Screening and Assessment of Mental Health Problems in The Perinatal Period: A Systematic Review and Meta-synthesis

**Journal:** Clinical Child and Family Psychology Review

**Authors:** Kerry Hozhabrafkan, Patricia Gooding, Anja Wittkowski, Sanaa Hyder and Sarah Peters

**Affiliation:** University of Manchester

**Corresponding Author Email:** Kerry.hozhabrafkan@postgrad.manchester.ac.uk
