# Supplementary material for: Women’s Experiences of Screening and Assessment of Mental Health Problems in the Perinatal Period: A Systematic Review and Meta-synthesis
Source: Clin Child Fam Psychol Rev. 2026 May 8;29(3):506–34. doi: 10.1007/s10567-026-00571-9 (PMC13562364; doi:10.1007/s10567-026-00571-9)
Supplement: Supplementary file 5 [file 10567_2026_571_MOESM5_ESM.docx]

**Table S4**

*Methodological Quality Assessment of Included Studies*

|  | **Study**  **(author & date)** | **Was there a clear statement of the aims of the research** | **Is a qualitative methodology appropriate** | **Was the research design appropriate to address the aims of the research** | **Was the recruitment strategy appropriate to the aims of the research** | **Was the data collected in a way that addressed the research issue** | **Has the relationship between researcher and participants been adequately considered** | **Have ethical issues been taken into consideration** | **Was the data analysis sufficiently rigorous** | **Is there a clear statement of findings** | **How valuable is the research** | **Quality Appraisal (total score)** | **Second appraiser** |
| --- | --- | --- | --- | --- | --- | --- | --- | --- | --- | --- | --- | --- | --- |
| 1 | **Shakespeare et al (2003)** | **Yes**  **(1)** | **Yes**  **(1)** | **Yes**  **(1)** | **Somewhat**  **(0.5)** | **Yes**  **(1)** | **Somewhat**  **(0.5)** | **Somewhat**  **(0.5)** | **Yes**  **(1)** | **Yes**  **(1)** | **Yes**  **(1)** | **8.5** | **8.5** |
| 2 | **Poole et al (2006)** | **Yes**  **(1)** | **Yes**  **(1)** | **Yes**  **(1)** | **Yes**  **(1)** | **Yes**  **(1)** | **No**  **(0)** | **Somewhat**  **(0.5)** | **Yes**  **(1)** | **Yes**  **(1)** | **Yes**  **(1)** | **8.5** | **8.5** |
| 3 | **Godderis et al (2009)** | **Somewhat**  **(0.5)** | **Yes**  **(1)** | **Yes**  **(1)** | **Yes**  **(1)** | **Yes**  **(1)** | **Somewhat**  **(0.5)** | **Yes**  **(1)** | **Somewhat**  **(0.5)** | **Yes**  **(1)** | **Yes**  **(1)** | **8.5** | **8.5** |
| 4 | **Armstrong & Small (2010)** | **Yes**  **(1)** | **Yes**  **(1)** | **Yes**  **(1)** | **Yes**  **(1)** | **Yes**  **(1)** | **No**  **(0)** | **Somewhat**  **(0.5)** | **Somewhat**  **(0.5)** | **Yes**  **(1)** | **Yes**  **(1)** | **8** | **8** |
| 5 | **Byatt et al (2013)** | **Yes**  **(1)** | **Yes**  **(1)** | **Somewhat**  **(0.5)** | **Yes**  **(1)** | **Yes**  **(1)** | **No**  **(0)** | **Somewhat**  **(0.5)** | **Yes**  **(1)** | **Somewhat**  **(0.5)** | **Yes**  **(1)** | **7** | **8** |
| 6 | **Darwin et al (2013)** | **Yes**  **(1)** | **Yes**  **(1)** | **Yes**  **(1)** | **Yes**  **(1)** | **Yes**  **(1)** | **Yes**  **(1)** | **Somewhat**  **(0.5)** | **Yes**  **(1)** | **Yes**  **(1)** | **Yes**  **(1)** | **9.5** | **10** |
| 7 | **Rollans et al (2013)** | **Yes**  **(1)** | **Yes**  **(1)** | **Yes**  **(1)** | **Yes**  **(1)** | **Yes**  **(1)** | **Yes**  **(1)** | **Somewhat**  **(0.5)** | **Yes**  **(1)** | **Yes**  **(1)** | **Yes**  **(1)** | **9.5** | **8.5** |
| 8 | **Coates et al (2015)** | **Yes**  **(1)** | **Yes**  **(1)** | **Somewhat**  **(0.5)** | **Yes**  **(1)** | **Yes**  **(1)** | **Somewhat**  **(0.5)** | **Yes**  **(1)** | **Yes**  **(1** | **Yes**  **(1** | **Yes**  **(1)** | **9** | **8** |
| 9 | **Mann et al (2015)** | **Yes**  **(1)** | **Yes**  **(1)** | **Yes**  **(1)** | **Yes**  **(1)** | **Yes**  **(1)** | **No**  **(0)** | **Somewhat**  **(0.5)** | **No**  **(0)** | **Yes**  **(1)** | **Yes**  **(1)** | **7.5** | **8.5** |
| 10 | **Darwin et al (2016)** | **Yes**  **(1)** | **Yes**  **(1)** | **Yes**  **(1)** | **Yes**  **(1)** | **Yes**  **(1)** | **No**  **(0)** | **Somewhat**  **(0.5)** | **Somewhat**  **(0.5)** | **Yes**  **(1)** | **Yes**  **(1)** | **8** | **8** |
| 11 | **Williams et al (2016)** | **Yes**  **(1)** | **Yes**  **(1)** | **Yes**  **(1)** | **Yes**  **(1)** | **Yes**  **(1)** | **No**  **(0)** | **Yes**  **(1)** | **Yes**  **(1)** | **Yes**  **(1)** | **Yes**  **(1)** | **9** | **8.5** |
| 12 | **Bayrampour et al (2017)** | **Yes**  **(1)** | **Yes**  **(1)** | **Somewhat**  **(0.5)** | **Yes**  **(1)** | **Yes**  **(1)** | **No**  **(0)** | **Somewhat**  **(0.5)** | **Yes**  **(1)** | **Yes**  **(1)** | **Yes**  **(1)** | **8** | **9.5** |
| 13 | **Evans et al (2017)** | **Yes**  **(1)** | **Yes**  **(1)** | **Yes**  **(1)** | **Yes**  **(1)** | **Yes**  **(1)** | **No**  **(0)** | **Somewhat**  **(0.5)** | **Yes**  **(1)** | **Yes**  **(1)** | **Yes**  **(1)** | **8.5** | **8.5** |
| 14 | **Doherty et al (2018)** | **Yes**  **(1)** | **Yes**  **(1)** | **Yes**  **(1)** | **Yes**  **(1)** | **Yes**  **(1)** | **No**  **(0)** | **Somewhat**  **(0.5)** | **Yes**  **(1)** | **Somewhat**  **(0.5)** | **Yes**  **(1)** | **8** | **8.5** |
| 15 | **Littlewood et al (2018)** | **Yes**  **(1)** | **Yes**  **(1)** | **Yes**  **(1)** | **Yes**  **(1)** | **Yes**  **(1)** | **No**  **(0)** | **Yes**  **(1)** | **Yes**  **(1)** | **Yes**  **(1)** | **Yes**  **(1)** | **9** | **9** |
| 16 | **Marsay et al (2018)** | **Yes**  **(1)** | **Yes**  **(1)** | **Yes**  **(1)** | **Yes**  **(1)** | **Yes**  **(1)** | **Yes**  **(1)** | **Yes**  **(1)** | **Yes**  **(1)** | **Yes**  **(1)** | **Somewhat**  **(0.5)** | **9.5** | **10** |
| 17 | **Nagle & Farrelly (2018)** | **Yes**  **(1)** | **Yes**  **(1)** | **Yes**  **(1)** | **Yes**  **(1)** | **Yes**  **(1)** | **No**  **(0)** | **Yes**  **(1)** | **Yes**  **(1)** | **Yes**  **(1)** | **Yes**  **(1)** | **9** | **8.5** |
| 18 | **Carlin et al (2019)** | **Yes**  **(1)** | **Yes**  **(1)** | **Yes**  **(1)** | **Yes**  **(1)** | **Yes**  **(1)** | **Yes**  **(1)** | **Yes**  **(1)** | **Yes**  **(1)** | **Yes**  **(1)** | **Yes**  **(1)** | **10** | **10** |
| 19 | **Skoog et al (2019)** | **Yes**  **(1)** | **Yes**  **(1)** | **Yes**  **(1)** | **Somewhat**  **(0.5)** | **Yes**  **(1)** | **Yes**  **(1)** | **Yes**  **(1)** | **Yes**  **(1)** | **Yes**  **(1)** | **Yes**  **(1)** | **9.5** | **10** |
| 20 | **Yapp et al (2019)** | **Yes**  **(1)** | **Yes**  **(1)** | **Yes**  **(1)** | **Yes**  **(1)** | **Yes**  **(1)** | **Yes**  **(1)** | **Yes**  **(1)** | **Yes**  **(1)** | **Yes**  **(1)** | **Yes**  **(1)** | **10** | **10** |
| 21 | **Doherty et al (2020)** | **Yes**  **(1)** | **Yes**  **(1)** | **Yes**  **(1)** | **Yes**  **(1)** | **Yes**  **(1)** | **No**  **(0)** | **Somewhat**  **(0.5)** | **Somewhat**  **(0.5)** | **Yes**  **(1)** | **Yes**  **(1)** | **8** | **8** |
| 22 | **Dyurich & Oliver (2020)** | **Yes**  **(1)** | **Yes**  **(1)** | **Yes**  **(1)** | **Yes**  **(1)** | **Yes**  **(1)** | **Yes**  **(1)** | **Somewhat**  **(0.5)** | **Yes**  **(1)** | **Yes**  **(1)** | **Yes**  **(1)** | **9.5** | **10** |
| 23 | **Field et al (2020)** | **Yes**  **(1)** | **Yes**  **(1)** | **Yes**  **(1)** | **Yes**  **(1)** | **Yes**  **(1)** | **Yes**  **(1)** | **Yes**  **(1)** | **Yes**  **(1)** | **Yes**  **(1)** | **Yes**  **(1)** | **10** | **10** |
| 24 | **Velloza et al (2020)** | **Yes**  **(1)** | **Yes**  **(1)** | **Yes**  **(1)** | **Yes**  **(1)** | **Yes**  **(1)** | **No**  **(0)** | **Somewhat**  **(0.5)** | **Yes**  **(1)** | **Yes**  **(1)** | **Yes**  **(1)** | **8.5** | **9.5** |
| 25 | **Williams et al (2016)** | **Yes**  **(1)** | **Yes**  **(1)** | **Yes**  **(1)** | **Yes**  **(1)** | **Yes**  **(1)** | **No**  **(0)** | **Yes**  **(1)** | **Yes**  **(1)** | **Yes**  **(1)** | **Yes**  **(1)** | **9** | **8.5** |
| 26 | **Hsieh et al (2021)** | **Yes**  **(1)** | **Yes**  **(1)** | **Yes**  **(1)** | **Yes**  **(1)** | **Yes**  **(1)** | **No**  **(0)** | **Yes**  **(1)** | **Yes**  **(1)** | **Yes**  **(1)** | **Yes**  **(1)** | **9** | **8.5** |
| 27 | **Kohlkoff et al (2021)** | **Yes**  **(1)** | **Yes**  **(1)** | **Yes**  **(1)** | **Yes**  **(1)** | **Yes**  **(1)** | **No**  **(0)** | **Somewhat**  **(0.5)** | **Yes**  **(1)** | **Yes**  **(1)** | **Yes**  **(1)** | **8.5** | **9** |
| 28 | **Chan et al (2022)** | **Yes**  **(1)** | **Yes**  **(1)** | **Yes**  **(1)** | **Yes**  **(1)** | **Yes**  **(1)** | **Yes**  **(1)** | **Yes**  **(1)** | **Yes**  **(1)** | **Yes**  **(1)** | **Yes**  **(1)** | **10** | **10** |
| 29 | **Mule et al (2022)** | **Yes**  **(1)** | **Yes**  **(1)** | **Yes**  **(1)** | **Yes**  **(1)** | **Yes**  **(1)** | **No**  **(0)** | **No**  **(0)** | **Yes**  **(1)** | **Yes**  **(1)** | **Yes**  **(1)** | **8** | **8** |
| 30 | **Pham et al (2022)** | **Yes**  **(1)** | **Yes**  **(1)** | **Somewhat**  **(0.5)** | **Yes**  **(1)** | **Yes**  **(1)** | **Yes**  **(1)** | **Yes**  **(1)** | **Yes**  **(1)** | **Yes**  **(1)** | **Yes**  **(1)** | **9.5** | **10** |
| 31 | **Fellmeth et al (2023)** | **Yes**  **(1)** | **Yes**  **(1)** | **Somewhat**  **(0.5)** | **Yes**  **(1)** | **Yes**  **(1)** | **Yes**  **(1)** | **Somewhat**  **(0.5)** | **Yes**  **(1)** | **Yes**  **(1)** | **Yes**  **(1)** | **9** | **10** |
| 32 | **Broberg et al (2024)** | **Yes**  **(1)** | **Yes**  **(1)** | **Yes**  **(1)** | **Yes**  **(1)** | **Yes**  **(1)** | **Yes**  **(1)** | **Yes**  **(1)** | **Yes**  **(1)** | **Yes**  **(1)** | **Yes**  **(1)** | **10** | **10** |
| 33 | **Dudeney et al (2024a)** | **Yes**  **(1)** | **Yes**  **(1)** | **Yes**  **(1)** | **Yes**  **(1)** | **Yes**  **(1)** | **No**  **(0)** | **Somewhat**  **(0.5)** | **Yes**  **(1)** | **Yes**  **(1)** | **Yes**  **(1)** | **8.5** | **8.5** |
| 34 | **Dudeney et al (2024b)** | **Yes**  **(1)** | **Yes**  **(1)** | **Somewhat**  **(0.5)** | **Yes**  **(1)** | **Yes**  **(1)** | **Yes**  **(1)** | **Yes**  **(1)** | **Yes**  **(1)** | **Yes**  **(1)** | **Yes**  **(1)** | **9.5** | **10** |
| 35 | **Yuill et al**  **(2024)** | **Yes**  **(1)** | **Yes**  **(1)** | **Yes**  **(1)** | **Yes**  **(1)** | **Yes**  **(1)** | **Somewhat**  **(0.5)** | **Yes**  **(1)** | **Yes**  **(1)** | **Yes**  **(1)** | **Yes**  **(1)** | **9.5** | **8.5** |
| 36 | **Hayward et al (2025)** | **Yes**  **(1)** | **Yes**  **(1)** | **Yes**  **(1)** | **Yes**  **(1)** | **Yes**  **(1)** | **Yes**  **(1)** | **Somewhat**  **(0.5)** | **Yes**  **(1)** | **Yes**  **(1)** | **Yes**  **(1)** | **9.5** | **10** |
| 37 | **Kanan et al**  **(2025)** | **Yes**  **(1)** | **Yes**  **(1)** | **Yes**  **(1)** | **Yes**  **(1)** | **Yes**  **(1)** | **Yes**  **(1)** | **Somewhat**  **(0.5)** | **Yes**  **(1)** | **Yes**  **(1)** | **Yes**  **(1)** | **9** | **10** |
| 38 | **Witcraft et al (2025)** | **Yes**  **(1)** | **Yes**  **(1)** | **Somewhat**  **(0.5)** | **Yes**  **(1)** | **Yes**  **(1)** | **Yes**  **(1)** | **Somewhat**  **(0.5)** | **Yes**  **(1)** | **Yes**  **(1)** | **Yes**  **(1)** | **9** | **9.5** |

**Level of agreement for high/medium/low quality = 34/38 = 89.47%**

High (>8-10) n=30

Medium (6-8) n=8

Low (0-5) n= 0

**Title:** Women’s Experiences of Screening and Assessment of Mental Health Problems in The Perinatal Period: A Systematic Review and Meta-synthesis

**Journal:** Clinical Child and Family Psychology Review

**Authors:** Kerry Hozhabrafkan, Patricia Gooding, Anja Wittkowski, Sanaa Hyder and Sarah Peters

**Affiliation:** University of Manchester

**Corresponding Author Email:** Kerry.hozhabrafkan@postgrad.manchester.ac.uk
